# Uncovering a multitude of stage-specific splice variants and putative protein isoforms generated along mouse spermatogenesis

**DOI:** 10.1186/s12864-024-10170-z

**Published:** 2024-03-20

**Authors:** Carlos Romeo-Cardeillac, María Fernanda Trovero, Santiago Radío, Pablo Smircich, Rosana Rodríguez-Casuriaga, Adriana Geisinger, José Sotelo-Silveira

**Affiliations:** 1 Laboratory of Molecular Biology of Reproduction, Department of Molecular Biology, Instituto de Investigaciones Biológicas Clemente Estable (IIBCE), 11,600 Montevideo, Uruguay; 2 Department of Genomics, IIBCE, 11,600 Montevideo, Uruguay; 3 Biochemistry-Molecular Biology, Facultad de Ciencias, Universidad de la República (UdelaR), 11,400 Montevideo, Uruguay; 4 Department of Cell and Molecular Biology, Facultad de Ciencias, UdelaR, 11,400 Montevideo, Uruguay; 5 Present Address: Boston Children’s Hospital, Harvard Medical School, Boston, MA USA

**Keywords:** Spermatogenesis, Transcriptome, Alternative splicing, lncRNAs, Testis

## Abstract

**Background:**

Mammalian testis is a highly complex and heterogeneous tissue. This complexity, which mostly derives from spermatogenic cells, is reflected at the transcriptional level, with the largest number of tissue-specific genes and long noncoding RNAs (lncRNAs) compared to other tissues, and one of the highest rates of alternative splicing. Although it is known that adequate alternative-splicing patterns and stage-specific isoforms are critical for successful spermatogenesis, so far only a very limited number of reports have addressed a detailed study of alternative splicing and isoforms along the different spermatogenic stages.

**Results:**

In the present work, using highly purified stage-specific testicular cell populations, we detected 33,002 transcripts expressed throughout mouse spermatogenesis not annotated so far. These include both splice variants of already annotated genes, and of hitherto unannotated genes. Using conservative criteria, we uncovered 13,471 spermatogenic lncRNAs, which reflects the still incomplete annotation of lncRNAs. A distinctive feature of lncRNAs was their lower number of splice variants compared to protein-coding ones, adding to the conclusion that lncRNAs are, in general, less complex than mRNAs. Besides, we identified 2,794 unannotated transcripts with high coding potential (including some arising from yet unannotated genes), many of which encode unnoticed putative testis-specific proteins. Some of the most interesting coding splice variants were chosen, and validated through RT-PCR. Remarkably, the largest number of stage-specific unannotated transcripts are expressed during early meiotic prophase stages, whose study has been scarcely addressed in former transcriptomic analyses.

**Conclusions:**

We detected a high number of yet unannotated genes and alternatively spliced transcripts along mouse spermatogenesis, hence showing that the transcriptomic diversity of the testis is considerably higher than previously reported. This is especially prominent for specific, underrepresented stages such as those of early meiotic prophase, and its unveiling may constitute a step towards the understanding of their key events.

**Supplementary Information:**

The online version contains supplementary material available at 10.1186/s12864-024-10170-z.

## Background

**Fig. 1:**
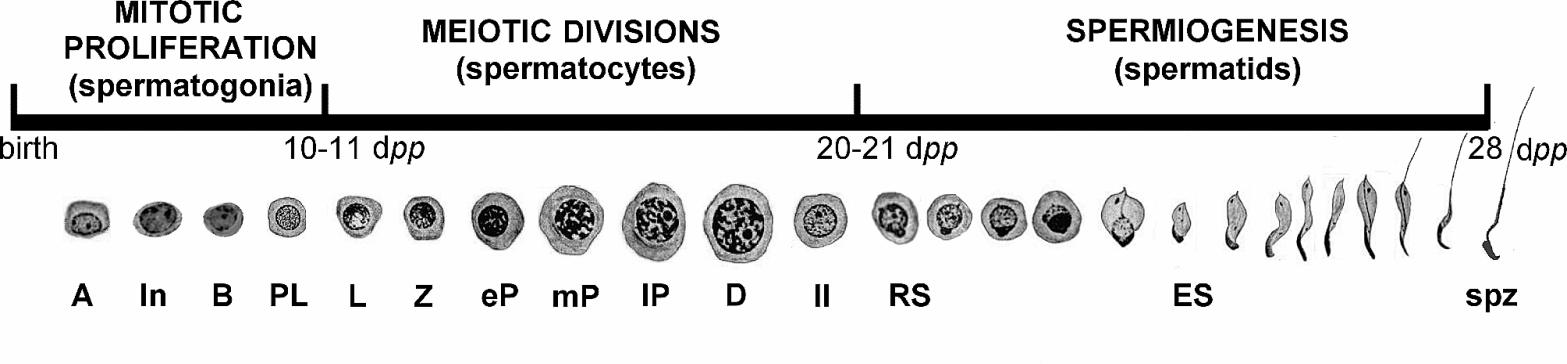
Schematic representation of the timing of spermatogenesis in the mouse. The three main phases of the process are shown. Emblematic stages are graphically represented under the timeline, and their postnatal timing of appearance is expressed as days *postpartum* (d*pp*). Cell types represented correspond to type A, intermediate (In), type B and preleptotenic (PL) spermatogonia; leptotenic (L), zygotenic (Z), early (eP), medium (mP) and late (lP) pachytenic primary spermatocytes, and diplotenic ones (D); secondary spermatocytes (II); round (RS) and elongating (ES) spermatids, and spermatozoa (spz). Adapted from reference 94, under the Creative Commons Attribution License (https://creativecommons.org/licenses/by/4.0/)

Spermatogenesis can be defined as the execution of three consecutive yet overlapping processes that take place in the male gonad, inside the seminiferous tubules. The first process is the mitotic proliferation and differentiation of spermatogonia (meiotic precursor cells), which go through different stages until they become primary spermatocytes and enter meiosis. The second phase is the meiotic divisions, during which spermatocytes (I and II, corresponding to the first and second meiotic divisions, respectively) halve their DNA content, resulting in haploid spermatids. Recombination of homologous chromosomes, which involves a meiotic-specific protein structure, the synaptonemal complex, is a hallmark of meiotic prophase I. The third phase, spermiogenesis, is the differentiation of round spermatids (i.e. the outcome of meiosis II) into sperm (Fig. 1). Along the latter, spermatids undergo dramatic changes, namely: the acquisition of a flagellum; nuclear elongation; loss of most cytoplasm; acrosome formation; reorganization of mitochondria; and the sequential replacement of most histones first by transition proteins and then by protamines, with the consequence of chromatin compaction and massive transcriptional silencing during late spermiogenic stages [1, 2].

Besides germline cells at their various differentiation stages, different somatic cell types coexist within the mammalian testes: Sertoli cells, which support and nourish the germline cells inside the seminiferous tubules; peritubular myoid cells; and different types of interstitial cells, including testosterone-producing Leydig cells, fibroblasts, macrophages, endothelial cells, innate lymphoid cells, and mesenchymal cells [3]. In total, the testis is composed of over 30 different cell types, which makes it an extremely complex and heterogeneous tissue.

Testicular tissue and cell complexity are also reflected at the transcriptional level. It has been shown that, in different mammalian species, the testes exhibit the highest transcriptomic complexity and diversity compared to other tissues, expressing the largest number of tissue-specific genes [4–6] and an overwhelming majority of long noncoding RNAs (lncRNAs) [6–11], as well as a panoply of short noncoding RNAs (piRNAs, miRNAs) [12–17]. Moreover, together with the brain, the testes have been reported to present the highest rate of alternative splicing (AS) [6, 18–21], which generates a huge number of testis-specific, temporally regulated RNA isoforms and protein variants [22, 23]. In accordance with this, the testis expresses a very large number of specific and strictly-regulated RNA-binding proteins [24–26], including many unique or differentially expressed (DE) splicing factors [20, 22, 27–29]. Furthermore, splicing defects have been associated with testicular pathologies [20, 22, 23, 29–32]. Interestingly, the complexity of the testicular transcriptome has been reported to mostly derive from primary spermatocytes and, particularly, round spermatids [6].

A number of studies have analyzed testicular transcriptomic diversity along spermatogenesis progression, and some of them included the identification and/or preliminary characterization of AS in mouse [6, 33–38], rat [39], and human [40]. However, only a very limited number of studies have addressed a more detailed analysis [34, 35, 38]. Moreover, they were mostly based on computational deconvolution approaches [35] or available data sets [38].

We have previously profiled the transcriptomic fluctuations along mouse spermatogenesis, both for coding transcripts [41] and for lncRNAs [42]. The input was highly purified stage-specific spermatogenic cell populations by flow-cytometry [43–45], thus constituting a solid basis for generating highly reliable information. Of particular interest, our analyses included purified early meiotic prophase cell populations, which have been very rarely included in transcriptomic studies. Here, we used our highly confident data to provide a comprehensive study of the transcriptomic diversity along spermatogenesis. Due to the purity of the populations, added to the depth of the libraries, we have been able to detect genes and isoforms that are lowly expressed and/or specific to scarce cell types, which had not been detected so far.

Overall, our results identify a high number of unannotated transcripts and splice variants, both coding and noncoding, which helps contribute to the understanding of testis complexity and functionality. This is particularly conspicuous for short, poorly studied stages, such as early meiotic prophase. Therefore, even for a genome as well characterized as that of the mouse, when it comes to specific stages of spermatogenesis, there is still much transcriptomic diversity to be described, including undisclosed stage-specific protein isoforms.

## Results

**Fig. 2:**
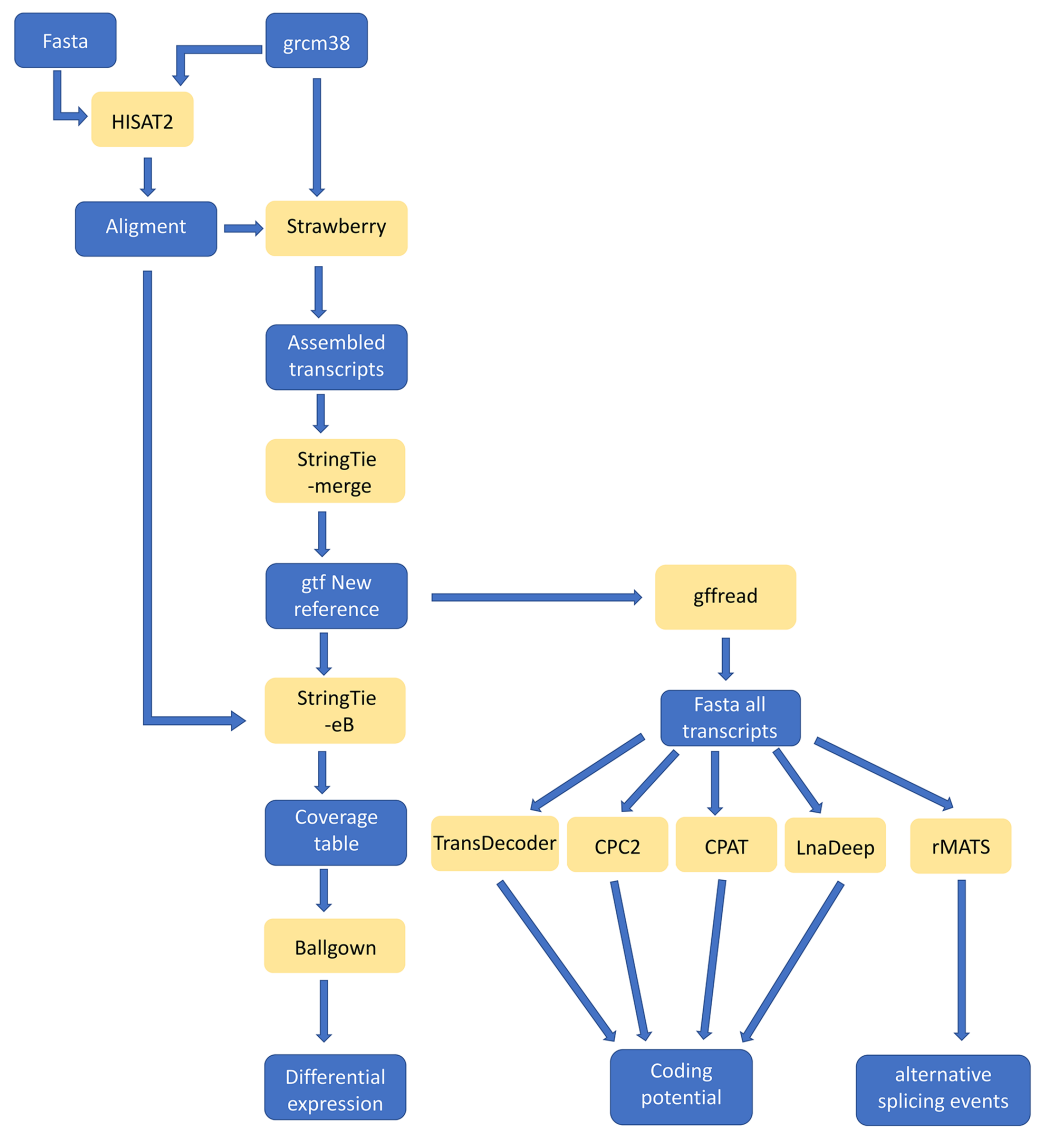
Flow chart of the followed bioinformatics pipeline. The data files are represented in blue, while the different employed software is represented in yellow

In previous reports, we have profiled the protein-coding and lncRNAs transcriptomes along mouse spermatogenesis, using isolated cell populations at different spermatogenic stages, and including a highly pure leptotene-zygotene (LZ) fraction [41, 42]. The latter allowed us to analyze early meiotic prophase, which is a scarce, short-lived stage, and therefore had been very poorly characterized at the molecular level. However, in those analyses we only studied annotated genes. Moreover, expressed genes were accounted for, but not splice variants. Here, we used our highly reliable raw data to identify unannotated expressed genes, stage-specific RNA species and unreported putative proteins, as well as to analyze AS and its variations along spermatogenesis in order to have a more complete idea of its real extension (see complete pipeline in Fig. 2).

A correlation matrix showed high reproducibility between biological replicas (Supplementary Figure S1). Besides, contrasting our data with those from another study, namely a single-cell RNA sequencing (scRNA-seq) of 20 different spermatogenic cell subtypes [37], rendered a good correlation despite the different methodologies employed in both studies (Supplementary Figure S2). Overall, our data is a very deep set of reads with robust reproducibility, and therefore it is useful to characterize even lowly expressed transcripts.

### Identification of unannotated coding and noncoding transcripts

**Fig. 3:**
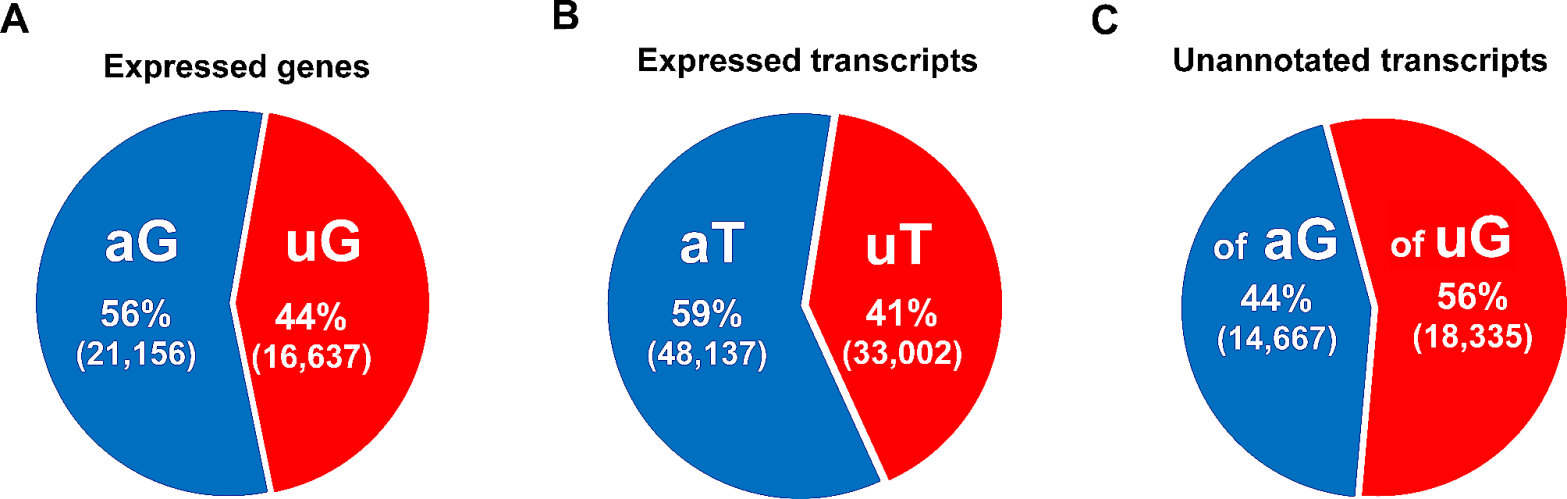
Genes and transcripts expressed in our lists. (**A**) Pie chart of annotated genes (aG: blue) and unannotated genes (uG: red) expressed in the four spermatogenic cell populations that passed all the filters. (**B**) Pie chart of annotated transcripts (aT: blue) and unannotated transcripts (uT: red) expressed in the four spermatogenic cell populations. (**C**) Pie chart showing the origin of the unannotated transcripts in our lists, either undisclosed splice variants of already annotated genes (of aG: blue), or transcripts arising from unannotated genes (of uG: red)

We applied strict cut-offs for downstream analyses (e.g. 10X coverage as minimum; 10 reads as minimum support per splice site; 10 reads as minimum per exon support). Under the selected conditions, we identified 37,793 testis-expressed genes that passed all the filters, of which 21,156 (56%) were already annotated in databases, and 16,637 (44%) were unannotated genes (Fig. 3A, and Supplementary Figures S3A and S3B). These 37,793 genes gave rise to 81,139 different transcripts (Supplementary Table S1). Of these transcripts, 48,137 (59%) were already annotated, while 33,002 (41%) were unreported transcripts (Fig. 3B, and Supplementary Figures S3A and S3C).

We then focused on the characterization of the 33,002 unannotated transcripts. Among them, we identified 14,667 (44%) as undisclosed splice variants of already known genes, while 18,335 (56%) corresponded to transcripts arising from regions of the genome for which there are no annotated transcripts (Fig. 3C, and Supplementary Figures S3A and S3D). This shows that there is still a very high number of testis-expressed genes and splice variants to be unveiled.

**Fig. 4:**
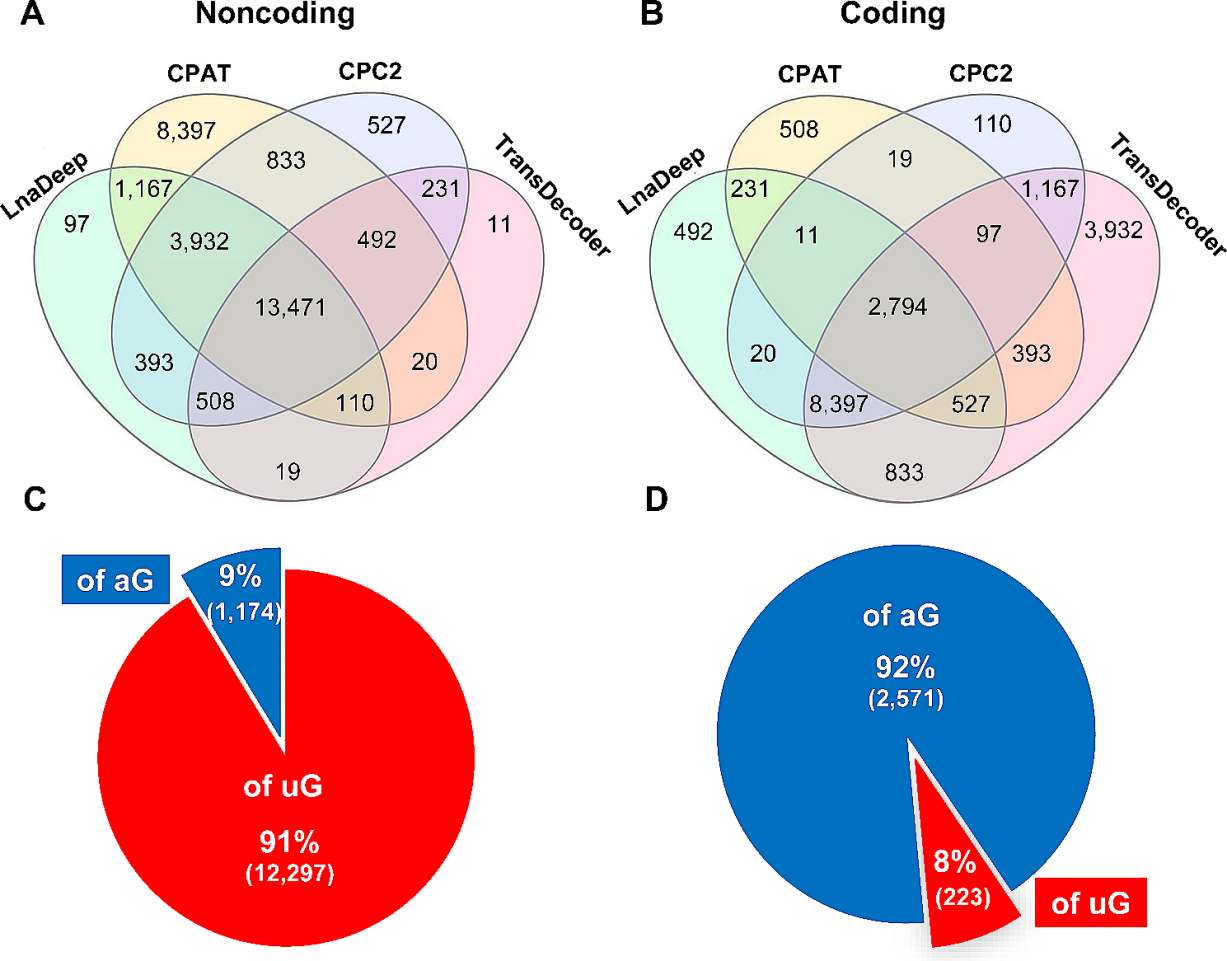
Coding potential of the unannotated transcripts. (**A**, **B**) Venn diagrams showing the analysis of the coding potential for the unannotated transcripts through four different software programs. (**C**, **D**) Pie charts of the unannotated noncoding and putative protein-coding transcripts that were coincidentally identified as such with the four programs, and classified into undisclosed splice variants of already annotated genes (of aG: blue) and transcripts of unannotated genes (of uG: red). **A**, **C**: noncoding transcripts; **B**, **D**: coding transcripts

Next, we analyzed the coding potential of the unannotated transcripts. For this purpose, we used four different software programs and only kept the results found in common among them (i.e., those transcripts for which all four programs coincide that they are, or are not, coding). The coincidence of the four programs identified 13,471 transcripts as noncoding (Fig. 4A), and 2,794 as coding (Fig. 4B). Therefore, most of the “novel” transcripts are noncoding. This is as expected since the coding genome has been much more characterized than the noncoding one. We note that our established criterion, which is very restrictive, excluded over half of the transcripts (e.g. if only three of the four programs coincided), but in turn allowed us to keep working with a highly reliable subset of transcripts in terms of their high or low coding potential.

Of the unannotated noncoding transcripts, the vast majority (12,297 transcripts, i.e. 91%) corresponded to unreported genes, namely lncRNAs, while less than 10% (1,174 transcripts) were undisclosed splice variants of already annotated noncoding genes (Fig. 4C).

Concerning the unannotated spermatogenic transcripts with highly reliable protein-coding potential, their identified number (2,794) is not negligible at all. Contrarily to the noncoding transcripts, the majority among them (2,571) corresponded to novel splice variants of already annotated genes, while less than 10% of the “novel” putatively coding transcripts, namely 223, were transcripts of unannotated genes (Fig. 4D, and Supplementary Table S2). Surprisingly, these 223 transcripts come from 191 yet unannotated genes. These results indicate that in the mouse genome there is still a significant number of putative protein-coding genes and AS coding isoforms that are expressed in spermatogenic cells, which have remained undetected so far.

We then focused on these 191 unannotated genes with high coding potential, and conducted functional analysis based on similarities with annotated genes from mouse and other species. Some of the similarities for encoded putative proteins were with ribosomal proteins, zinc finger proteins, and with putative Cilia and Flagella-Associated Protein 92 (MSTRG.30402.2; BlastX match to human FLJ43738). Besides, a relatively large subset (over half of the genes) corresponded to the products of retroposons and, to a lesser extent, integrated viruses. Finally, for 29 of these 191 genes (corresponding to 49 transcripts), no known probable function was associated (see Supplementary Table S2).

### Expression of the newly identified transcripts along the different spermatogenic stages

**Fig. 5:**
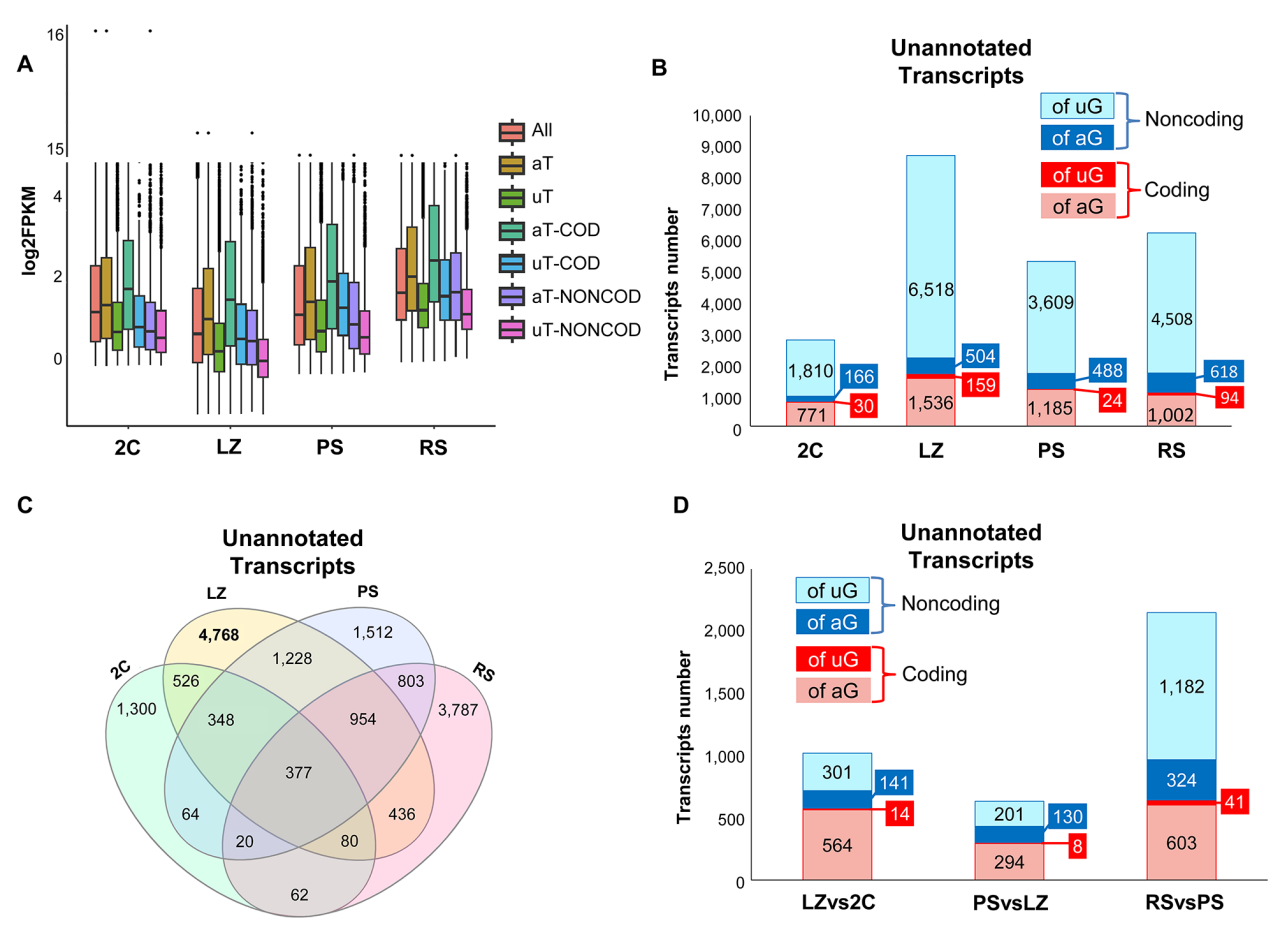
Distribution of the transcripts in the four testicular cell populations. (**A**) Box plot of expression levels (log2FPKM) of all detected transcripts. All: all detected transcripts; aT: annotated transcripts; uT: unannotated transcripts; aT-COD: annotated coding transcripts; uT-COD: unannotated coding transcripts; aT-NONCOD: annotated noncoding transcripts; uT-NONCOD: unannotated noncoding transcripts. (**B**) Unannotated transcripts that were coincidentally identified as such with the four programs for coding potential analysis (and depicted in Fig. 4), distributed according to their expression in each of the four testicular cell populations. Transcripts are categorized into coding or noncoding, and transcripts of unannotated genes (of uG) or splice variants of already annotated genes (of aG). Note that many transcripts may be expressed in more than one stage. (**C**) Venn diagram indicating the distribution of the transcripts represented in B, in the four testicular cell populations. (**D**) DE coding and noncoding transcripts between pairwise sample comparisons of the four populations in chronological order. Of uG and of aG denote the same as in B

As a next step, we analyzed the expression of the newly identified transcripts distributed by cell population. In the first place, we compared the expression levels of the unannotated transcripts with those previously annotated in Ensembl database, for each of the four cell populations. Overall, the median expression levels of the unannotated transcripts - both coding and noncoding - were lower than those of the annotated ones, and this turned out to be valid for all the cell populations (Fig. 5A). This may help explain why these transcripts had not been detected so far. Additionally, noncoding transcripts showed lower expression levels than coding ones for all cell populations (see Fig. 5A), which is in agreement with previous reports that indicate that the expression levels of lncRNAs are, in general, lower than those of mRNAs [9, 11].

Concerning the number of transcripts in each of the cell populations, interestingly, the largest contribution of the unannotated transcripts was on behalf of the LZ stage, both for noncoding and for coding transcripts (Fig. 5B). Furthermore, 55% of the unannotated LZ-expressed transcripts were exclusive of LZ (Fig. 5C, and Supplementary Figure S4A; see also Supplementary Figure S4B). In this regard, when we particularly looked at the unannotated coding genes, strikingly, 159 out of the 191 identified were expressed in LZ, and almost half (92 genes) were exclusive of LZ (see Supplementary Table S2). This led us to ask whether this would be the reflect of a greater number of transcripts expressed in LZ in general. Indeed, when we compared the total number of transcripts (both annotated and unannotated together) between the four testicular cell populations, LZ presented the highest number (Supplementary Figure S4C). Transcript saturation analysis including the data from the present study as well as from a previous one [41] showed that all the cell populations reached saturation (Supplementary Figure S5A). Moreover, the transcript expression histograms among all the four populations presented a similar distribution (Supplementary Figure S5B), thus helping validate the results. Altogether, these analyses confirm that the results are not an artifact of either the technique or the conducted analysis.

On the other hand, LZ was, in general, the stage with the lowest expression levels for all types of transcripts (both coding and noncoding, either annotated or not), while round spermatids (RS) transcripts exhibited the highest overall expression levels (see Fig. 5A). Thus, LZ expresses the largest number of stage-specific transcripts, although these are, in general, expressed at comparatively lower levels.

Next, we analyzed the differential expression of the newly identified transcripts among pairwise comparisons along the progress of the spermatogenic wave (log2 FC ≥ │2│, FDR < 0.05). We observed the highest number of DE unannotated transcripts that passed our established criteria at the pachytene spermatocytes (PS) - to - RS transition (Fig. 5D), and this is especially so for noncoding transcripts. This indicates that the transition from meiotic prophase to spermiogenesis involves the differential expression of a high number of genes and splice variants. Besides, this result is also reflecting the fact that, although as stated above, LZ expresses the highest number of exclusive unannotated transcripts, many of them are expressed at low levels, and therefore they do not pass our strict criterion for the definition of differential expression.

### Characterization of spermatogenic-specific AS

**Fig. 6:**
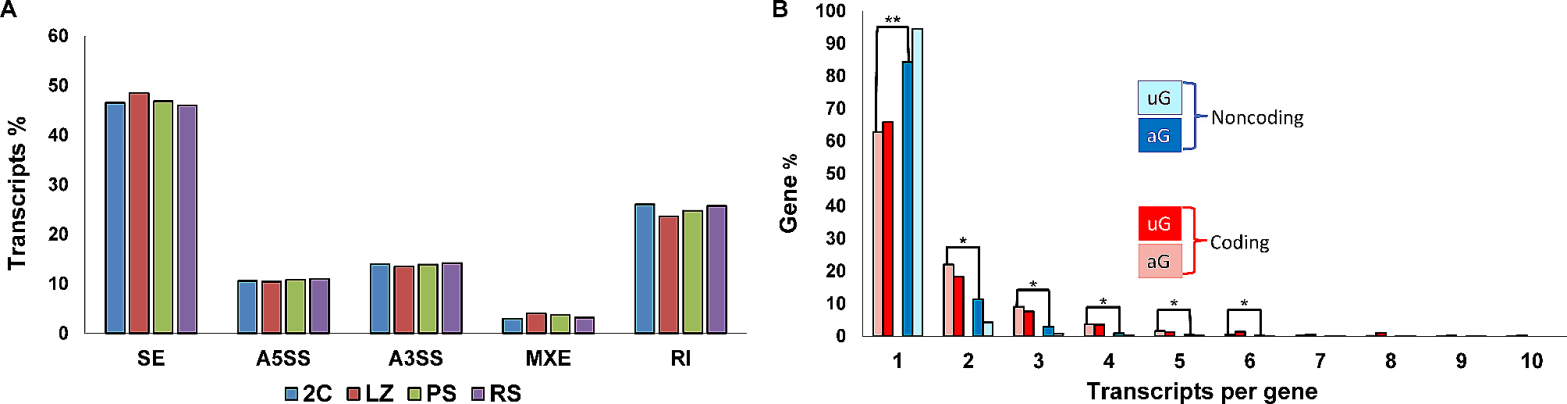
Analysis of spermatogenic-specific alternative splicing (AS). (**A**) Bar graph representing the distribution of different AS types (percentage) along the four testicular cell populations. SE: skipping exon; A5SS: alternative 5´ splice site; A3SS: alternative 3´ splice site; MXE: mutually exclusive exons; RI: retained intron. (**B**) Classification of the expressed genes (coding and noncoding), according to their number of splice variants in our lists. The data are presented as percentage of the total. Only genes with 1 to 10 expressed splice variants were considered. uG: unannotated genes; aG: annotated genes. ** *p* < 10^− 10^; * *p* < 0.01

We proceeded to further characterize the identified splice variants in our lists (both annotated and unannotated). In first place, we analyzed the different AS types in the four testicular cell populations by means of rMATS, and using the different AS categories defined by the software, i.e.: skipping exon (SE), alternative 5´ splice site (A5SS), alternative 3´ splice site (A3SS), mutually exclusive exons (MXE), and retained intron (RI). There were no significant enrichments when comparing AS events among the analyzed stages. On the other hand, SE and RI were the most abundant AS types, followed by A3SS, A5SS and MXE, in that order, in the four testicular cell populations (Fig. 6A).

Then, we studied the number of splice variants per gene. Clearly, we found that most splicing isoforms are generated by coding genes. Contrarily, noncoding genes, in general, have a lower number of transcripts per gene (Fig. 6B). Indeed, while about 60% of the coding genes express only one transcript, between 85% and 95% of the noncoding genes present only one transcript (*p* < 10^− 10^). Likewise, the number of genes with two or more splicing isoforms was higher for coding than for noncoding genes (*p* < 0,01). Basically, both annotated and unannotated genes behaved similarly in this regard, and this statement is valid both for coding and for noncoding transcripts (see Fig. 6B). This shows the reliability of our data, as there is no reason to suspect that the annotated and unannotated transcripts should behave differently. Furthermore, we note that although we are here showing the grouped results of the four testicular cell populations, there were no significant differences when the four populations were analyzed separately (not shown).

### In-depth analysis of representative newly identified putative protein-coding splice variants

As stated above, the expression levels of the unannotated transcripts were, in general, lower than those of annotated ones (see Fig. 5A). Notwithstanding this, it is worth mentioning that some of the newly identified transcripts presented very high expression levels, with some AS isoforms being much more highly expressed than the already annotated ones (see Supplementary Table S1, and examples mentioned below).

**Fig. 7:**
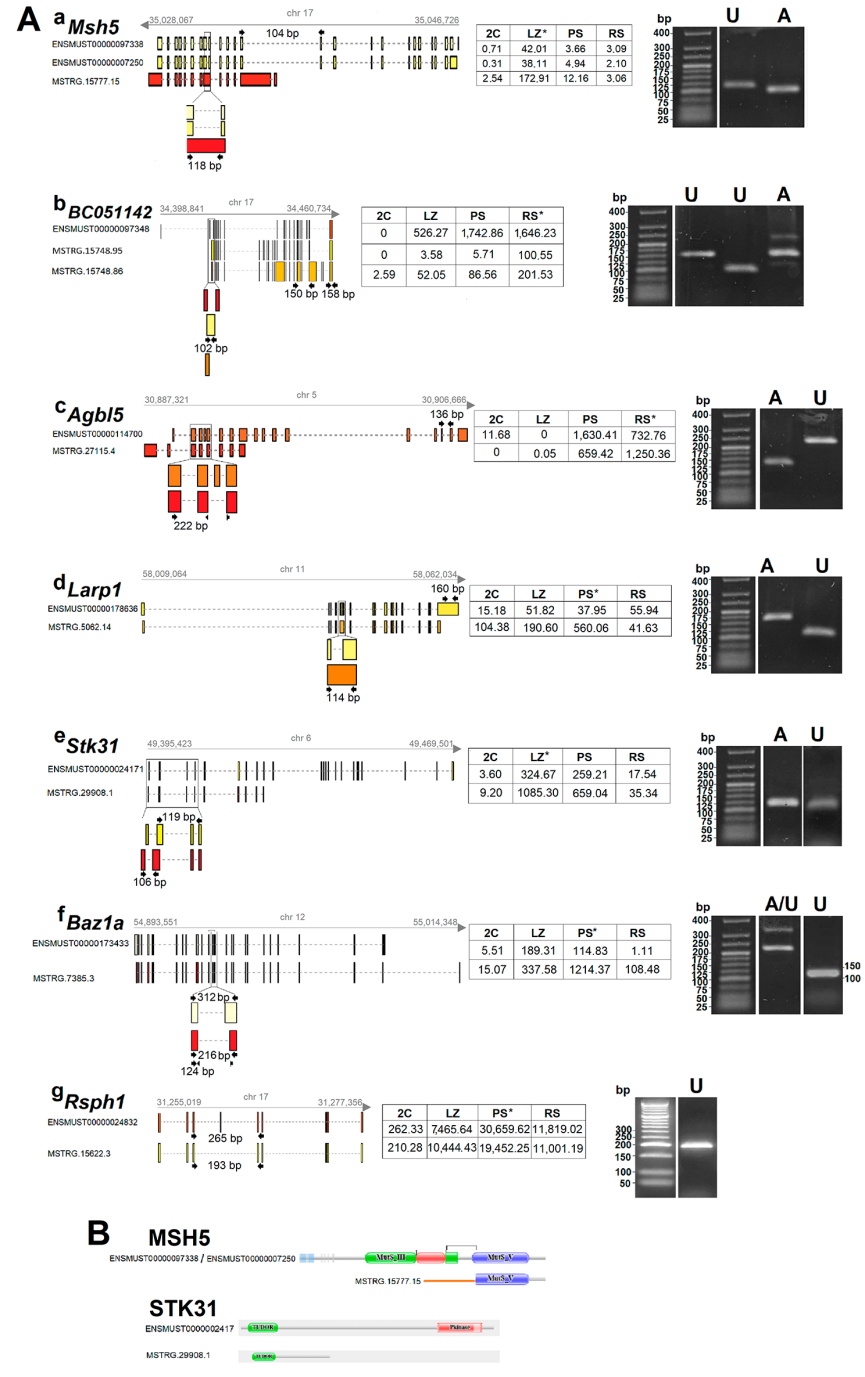
RT-PCR confirms the expression of different examples of selected putatively protein-coding splice variants. (**A**) Schematic representations of the splice variants (annotated and newly identified), and agarose gels showing their RT-PCR amplification. Ensembl annotations are depicted on the left as “ENSMUST” followed by the corresponding Ensembl numberings. Unannotated transcript isoforms are depicted with the label “MSTRG”. The designed primer sets for the amplification of either the annotated or the unannotated isoforms are shown for each case (arrows), together with the expected PCR product sizes (bp). The gray arrow above each diagram indicates transcription direction. Genomic location, as well as chromosome number, are indicated in each case. Whenever necessary, magnified insets are shown below each representation for better visualization of the amplified regions. A table with the coverage of the annotated and unannotated transcripts in the four cell populations is included in each case. The asterisks indicate the cell population in which the unannotated isoform was most highly expressed. In the agarose gels, A stands for the annotated splice variants, and U for the unannotated ones. Gels have been cropped for the sake of clarity (original agarose gels are presented as Supplementary Figure S7). (**a**) *Msh5* splice variants that encode the canonical 833 amino acids protein (yellow), and an unannotated splice variant encoding a putative 362 residues isoform (red). (**b**) *BC051142* most highly expressed annotated variant (red), and two unannotated putatively coding variants with a differential expression pattern along spermatogenesis (one is mostly differential of spermiogenesis, while the other progressively increases from early meiotic prophase to spermiogenesis; yellow and orange, respectively). In the lane corresponding to the annotated variant, two additional faint bands can be observed, most probably corresponding to the amplification of a couple of weakly expressed isoforms (due to the extremely high number of isoforms detected for this gene, it was not possible to design primer combinations to exclusively recognize only one variant). (**c**) *Agbl5* canonical transcript (orange), which encodes an 846 residues protein, and a selected unannotated variant (red) encoding a putative much shorter isoform of 412 amino acids. (**d**) The chosen *Larp1* unannotated splice variant (orange) encodes a putative not reported protein isoform of 760 amino acids, unlike the canonical one (yellow), whose encoded protein is 1,072 residues long. As shown, in PS the expression levels of the new variant are fifteen-fold higher than those of the canonical one. (**e**) The unannotated *Stk31* isoform we chose for confirmation (red) encodes a shorter variant that is much more highly expressed than the canonical one (yellow). The comparatively poorer amplification of the unannotated variant is due to the fact that the region did not allow the design of a good pair of primers. (**f**) Representation of an annotated *Baz1a* isoform (light yellow), and the unannotated splice variant (red), which is much more highly expressed all along spermatogenesis, upregulates in PS, and directs the synthesis of a shorter protein. In this case, amplification was performed with a primer set that simultaneously amplifies a region of both the annotated (312 bp) and unannotated (216 bp) variants. The annotated isoform is poorly amplified, presumptively due to its competition with the newly identified one, which is expressed at much higher levels (see table). Besides, a band corresponding to the amplification product with a primer set that only recognizes the unannotated isoform is shown to the right. (**g**) *Rsph1* was chosen as an example of a novel coding isoform generated through exon-skipping (yellow, while the canonical isoform is represented in red). Although the primer set was intended to amplify the annotated variant as well, yielding a larger, 265 bp band, the latter was not detected most probably because of its competition with the unannotated isoform. **B)** Representative schematic diagrams of two of the canonical and unannotated putative protein isoforms, to exemplify the differences between them. MSH5: The orange line in the “novel” isoform represents the first 133 amino acids, which are completely different from those of the canonical protein. STK31: While both isoforms present a Tudor domain, the predicted variant would lack the protein-kinase domain, which is essential for its function as a serin-threonine kinase in the canonical isoform

We chose seven examples of these unannotated splice variants to confirm the discovery through RT-PCR (Fig. 7A), using the following criteria: (i) Annotated coding genes that would have a high number of expressed splice variants in our lists; (ii) That at least one of the splice variants would code for an unannotated putative protein isoform; (iii) That the putative novel protein isoform would be significantly different (e.g. with different protein domains) from the annotated one(s); (iv) That the putative novel isoform would exhibit a relatively high expression level in at least one of the analyzed spermatogenic stages; and (v) That the annotated gene would have an interesting described function (e.g. testis-related), or would present a specific trait that we considered particularly interesting.

One of the selected genes was *MutS homologue 5* (*Msh5*), which is upregulated in LZ, and in mouse directs the synthesis of an 833 amino acids protein (Fig. 7A,a). We have identified fifteen unannotated splice variants for this gene (see Supplementary Table S1), and chose for confirmation one of them, which is also upregulated in LZ but much more highly expressed than the canonical one (see Fig. 7A,a). The selected transcript variant, which is generated through an alternative start site and a combination of all the above described AS mechanisms (i.e. SE, A5SS, A3SS, MXE, RI), encodes a putative shorter, 362 residues protein, containing an identical carboxyl-terminal region to that of the canonical protein, but a completely different amino-terminal region (see Fig. 7B).

We also chose *BC051142*, which ranked among the genes with the highest number of splice variants in our lists, as we detected 25 RNA isoforms expressed along spermatogenesis (when we used slightly less restrictive parameters, the number of expressed RNA isoforms for this gene raised to 103 splice variants). While there are eight putatively coding isoforms annotated in Ensembl, our analysis unveils the existence of at least nine additional unannotated protein-coding isoforms for this gene. None of the isoforms was detected in the 2 C cell population (i.e. somatic testicular cells and spermatogonia), and the expression of all of them starts in LZ, raising along spermatogenesis progress (Supplementary Table S1). In particular, we selected two unannotated putative protein-coding isoforms (Fig. 7A,b), for confirming their existence.

We also chose *ATP/GTP Binding Protein Like 5* (*Agbl5*), for which we have found several unannotated coding splice variants that are expressed at different levels along spermatogenic stages (see Supplementary Table S1). In particular, we selected for confirmation a very highly expressed variant that attains its expression peak in RS and encodes a putative 415 amino acids protein, unlike the canonical isoform whose highest expression level is in PS, and whose protein product is 846 residues long (Fig. 7A,c).

Additionally, we chose *La-Related Protein 1* (*Larp1*), *Serine-Threonine Kinase 31* (*Stk31*), *Bromodomain Adjacent to Zinc Finger Domain 1a* (*Baz1a*), and *Radial Spoke Head Component 1* (*Rsph1*). For all these genes, we have selected for confirmation unannotated highly expressed splice variants (Fig. 7A,d-g) that encode putative proteins that significantly differ from the annotated ones. In most cases, these novel variants are much more highly expressed than the canonical ones (see Fig. 7A,d-f). At least for some of them, their protein products would lack key domains (Fig. 7B), suggesting that these putative isoforms would accomplish different roles than the canonical ones.

We have been able to confirm the existence of all the chosen splice variants (see Fig. 7A,a-g), which further validates the results from our lists and shows the high reliability of our data concerning the identification of unannotated spermatogenic isoforms.

## Discussion

### The RNAseq analysis of highly pure stage-specific spermatogenic cell populations reveals a high number of undisclosed transcripts in early meiotic prophase

Different reports have indicated that the testis has a particularly complex transcriptome [2, 6], with AS significantly contributing to its complexity [20, 22, 23, 29, 34]. Moreover, it is known that proper stage-specific AS is critical for successful spermatogenesis [20, 22, 23, 29–32, 46]. However, due to the heterogeneous composition of the testis, most likely an important number of cell type-specific RNA isoforms fall below the detection limits when whole testes or poorly purified cell types, are employed for transcriptome studies. Moreover, despite scRNA-seq allows to study the transcripts of individual cells, which has recently helped improve the understanding of spermatogenesis [47], it is important to take into account that scRNA-seq libraries are lower in depth than those for bulk sequencing, which does not allow the detection and assembly of low expression transcripts. Here, the use of highly purified stage-specific spermatogenic cell populations, added to the depth of the sequencing libraries, allowed us to detect a high number of yet unannotated genes and AS transcripts, hence showing that the transcriptomic diversity of the testis is considerably higher than previously reported.

The LZ cell population showed the majority of unannotated splice variants. This can be partly explained by our finding that they present lower overall expression levels compared to those of the other testicular cell populations, which would be in agreement with early reports that suggested the existence of low global transcription levels in mouse testes during early meiotic prophase [48–50].

Another important factor that surely contributed to hamper the previous detection of many LZ transcripts, is that these stages are very short and difficult to obtain as isolated cell populations, and therefore they have been rarely used in transcriptomic studies in comparison to other spermatogenic stages such as medium/late meiotic prophase and spermiogenesis [41, 42]. Besides, it is reasonable to think that, due to the scarceness of these cell types, specific transcripts of them may have become diluted among those of the most abundant cell types in whole testes transcriptomes. Remarkably, 159 out of the 191 newly identified putatively coding genes are expressed in LZ spermatocytes, and almost half of them are exclusive of LZ; we can reason that they may have gone unnoticed so far precisely because they encode LZ-specific products. Of note, surely something similar happens with scanty cell types in other heterogeneous tissues, where a high number of specific transcripts must still be undetected.

Beyond the fact that LZ stages presented the largest number of unannotated transcripts among all the analyzed cell populations, they also showed the highest number of transcripts considering both those annotated and unannotated together. In fact, our results are in line with a scRNA-seq study that has suggested that early spermatogenic stages express a higher number of genes, while later stages tend to concentrate a higher fraction of their transcripts on a narrower set of genes [3]. We propose that this high number of LZ-genes and isoforms could be required to accomplish the unique events that take place during early meiotic prophase. Noteworthy, the molecular groundwork of such events is largely unknown: we still do not really understand the role of bouquet formation, neither how homologous chromosomes recognize each other. In this scenario, the identification of all these unannotated genes and splice variants (both coding and noncoding), may represent a step forward toward the understanding of these essential processes and how they are regulated.

### A large amount of still unannotated spermatogenic lncRNAs

The analysis of the coding potential of the unannotated transcripts, indicated that the highest number of them are noncoding (see Fig. 4). This makes sense as research regarding lncRNAs is much more recent than that of coding genes, and indicates that, when it comes to lncRNAs, we have only seen the tip of the iceberg, and there is still a high number of them to be discovered.

In relation to this, in a previous study, while attempting to conduct conservation analysis between spermatogenic lncRNAs of mouse and human, we found that for several lncRNAs from one species there were homologous DNA sequences in the other, but a cognate lncRNA was not annotated [42]. Although certainly this may be evidencing species-specific expression differences - which agrees with the fact that the expression patterns of lncRNAs are less conserved than those of coding genes [11] - this result may be also reflecting, at least in part, the incompleteness of the annotation of lncRNAs.

We have detected most of the DE lncRNA transcripts at the transition from meiosis (PS) to spermiogenesis (RS). This agrees with our previous observation that most of the differential expression of lncRNA genes along spermatogenesis takes place in spermiogenesis [42], and extends this result to splice variants.

The high numbers of unannotated spermatogenic lncRNAs we have identified, which add to the much higher amount of already annotated lncRNAs in male germ cells than in any other analyzed tissues and cell types [6–11], may be partly interpreted as a consequence of the relaxed chromatin of meiotic and post-meiotic cells, but also for the high levels of post-transcriptional regulation that are present in these cells (see next section).

A particular characteristic we found for noncoding genes was a lower number of transcripts per gene in comparison to protein-coding ones, thus indicating that noncoding genes tend to have less AS isoforms. The latter is in consonance with some earlier reports that indicated that the splicing of lncRNAs was less efficient than that of mRNAs [9, 51]. Besides, this is also in line with our results and those of other groups, which showed that lncRNAs tend to be shorter and have less exons than mRNAs [7, 9, 42], adding to the conclusion that lncRNAs are, in general, less complex than mRNAs.

### The number of unannotated transcripts and splice variants reinforces the concept of the high transcriptomic complexity of meiotic and post-meiotic cells

The meiotic and post-meiotic cell populations presented a higher number of unannotated transcripts compared to the 2 C cell population. This comparatively low number of the 2 C population may be reflecting the already known fact that meiotic and post-meiotic cells have extremely complex transcriptomes [6].

The widespread transcriptome complexity of male meiotic and post-meiotic cells has been proposed to be a consequence of their permissive chromatin state, which in turn results from the extensive chromatin remodeling that, due to histone replacement, takes place during these stages [6]. In this regard, we can speculate that at least part of the high number of unannotated transcripts that we found in meiotic and post-meiotic cells represents promiscuous transcription. In connection with this, while this manuscript was under review, a paper by Peters and collaborators [52] also showed a high number of novel unannotated transcripts in mouse male germ cells. Remarkably, the authors analyzed whether the expression of the high number of discovered transcripts could be influenced by repetitive elements in a cell type-specific manner, and found no evidence supporting that hypothesis.

On the other hand, the extensive transcriptome diversity of meiotic and especially of post-meiotic cells is also viewed as part of a strategy to regulate protein synthesis in the transcriptionally inert elongating and elongated spermatids. The need to have all the transcripts available to be translated in a timely fashion led to the development of diverse post-transcriptional regulatory mechanisms - some of which are unique to spermatocytes and RS - to accomplish the strict regulation requirements [1, 2, 25, 53]. In turn, these post-transcriptional regulation mechanisms most probably require a high amount of regulatory RNAs. In fact, although a large proportion of the spermatogenic lncRNAs are probably nonfunctional, at least for some of them, their importance for the regulation of spermatogenesis progression and fertility preservation, is being demonstrated [2, 54–63].

In summary, our results indicate that the transcriptomic complexity of spermatogenic cells is even higher than previously reported, and reinforces the concept that AS is particularly prominent for meiosis and spermiogenesis.

### Characterization of AS patterns reveals previously unknown interesting splice variants

The analysis of our RNAseq data showed SE to be the most abundant AS type, followed by RI, for the four cell populations. This is in agreement with the results shown by Li et al. in a reanalysis study of repository-available data (of mention, early meiotic prophase was not included in that study) [38]. Our results also agree with those of Naro et al. [53]., who found RI as one of the most represented regulated AS patterns in the trans-meiotic differentiation of male germ cells. Noteworthy, they observed that RI events were upregulated in spermatocytes compared to spermatids, suggesting that intron retention represents a modality of nuclear retention of transcripts in meiosis, for their timely translation in inactive post-meiotic germ cells [53]. Although we did not detect significant differences regarding RI between the four cell populations, it must be noted that these results are not comparable, as we only analyzed the prevalence of the diverse AS categories in the different spermatogenic cell populations, but not differentially regulated splicing events.

We also detected some unannotated splice variants with much higher expression levels than the annotated ones. In many cases, they may have gone unnoticed because they are highly expressed in a specific stage, which is often poorly represented (i.e., LZ). More important, for the newly identified AS transcripts with high coding potential, despite the limitation that the confirmation of the existence of their protein products is pending, most probably at least part of them encode unnoticed testis-specific protein isoforms. We can hypothesize that, at least some of them, have “novel” testis-specific functions. A key aspect in understanding the physiological validity of the discovery of interesting unannotated splice variants is that we were able to detect them using an alternative approach to RNA-seq, i.e. RT-PCR. Remarkably, they all represent examples of previously undisclosed, putative protein-coding isoforms that are DE along spermatogenic stages, and whose canonical proteins, in most cases, are known to play essential roles in spermatogenesis. In some cases, the putative unannotated protein isoforms would lack important domains.

An interesting example of the above is the undisclosed isoform we detected for *Msh5.* MSH5 is a meiotic-specific mismatch repair protein involved in homologous recombination [64] that has proved to be essential for meiotic progression [65]. The novel isoform, whose transcript is abundant in LZ, would have an incomplete ATPase domain that is required for double strand breaks repair [66], thus suggesting that this unannotated isoform could be accomplishing a different role during meiotic prophase.

Another, curious, example is *BC051142*,a gene that according to NCBI database is highly testis-specific (see https://www.ncbi.nlm.nih.gov/gene/?term=BC051142), and whose human homolog, *Testis Expressed Basic Protein 1* (*TSBP1*), has been associated with hypogonadism (https://www.genecards.org/cgi-bin/carddisp.pl?gene=TSBP1&keywords=BC051142). However, despite it encodes a high number of spermatogenic-specific different putative protein isoforms, its function is still unknown. Therefore, it constitutes an excellent example to illustrate the enormous variability that exists throughout spermatogenesis, and all that remains to be unveiled.

Concerning *Agbl5*, it is a highly testis-biased gene (https://www.ncbi.nlm.nih.gov/gene/?term=agbl5+mus+musculus) that encodes a metallocarboxypeptidase involved in tubulin deglutamylation, which is essential for the formation of functional sperm. It has been shown that AGBL5 (also known as CCP5) is necessary for the integrity of sperm flagella and for other microtubule-based functions during spermatogenesis [67, 68]. Although various splice variants have been reported, at least one of them even with apparently distinct properties [67], according to our findings several other unannotated coding splice variants expressed along spermatogenesis would exist.

*Larp1* encodes an RNA-binding protein that regulates the translation and stability of mRNAs for ribosomal proteins and translation factors downstream of TORC1 complex [69, 70], and is most highly expressed in the testis compared to other tissues (https://www.ncbi.nlm.nih.gov/gene/73158). *Stk31* is a testis-biased gene (https://www.ncbi.nlm.nih.gov/gene/77485) that encodes a serine-threonine kinase with a Tudor domain, which is preferentially localized in germinal granules of spermatocytes and acrosomal cap of spermatids, interacting with MIWI protein [71]. Besides, it has been shown to be a cancer/testis antigen highly expressed in several types of cancers [72–74]. *Baz1a* is highly [75] and dynamically expressed during spermatogenesis [76], and encodes a defining subunit of an ATP-dependent chromatin remodeler complex essential for proper spermatogenic gene expression and fertility in mouse [75]. *Rsph1*, whose expression is testis-restricted [77] (see https://www.ncbi.nlm.nih.gov/gene/22092), directs the synthesis of a component of radial spokes head of cilia and sperm flagella [78], and mutations in this gene have been related to fertility problems in humans, resulting in primary ciliary dyskinesia and motility alterations of cilia and sperm [79]. For all these genes, the newly identified putative protein isoforms would differ significantly from the canonical ones. STK31 is an example of this: while the known protein has a Tudor domain and a protein-kinase domain that is essential for its function as a serin-threonine kinase, the predicted variant would lack the latter, thus suggesting that it should play a different role.

## Conclusions

In this work, we generated a great amount of highly reliable information about gene expression along spermatogenesis, from pure flow sorted stage-specific mouse spermatogenic cell populations. Our results reveal a high number of yet unannotated spermatogenic lncRNAs, undisclosed splice variants of coding genes, and even some unannotated protein-coding genes. At least part of the newly identified splice variants encodes putative isoforms of important spermatogenic proteins. Besides, we have delved into the characterization of spermatogenic alternative splicing. Importantly, the largest number of spermatogenic stage-specific unannotated transcripts and splice variants are expressed during early meiotic prophase, a stage that has been scarcely studied in former transcriptomic analyses. We propose that these may be related to the unique and complex processes that take place during these stages.

Overall, this study shows that testicular transcriptomic diversity is considerably higher than previously reported. A general conclusion we can draw is that not only a great deal of existing variability in terms of spermatogenic non-coding RNAs and stage-specific protein variants is still to be revealed, but we do not even know the exact number of coding genes yet, even in a model as studied as the mouse.

## Methods

### Raw data

The raw data employed in this study came from stranded RNAseq libraries of testis-specific cell populations representative of landmark stages along mouse spermatogenesis, obtained through flow sorting [42] (SRA repository access number PRJNA548952). The cell populations were: 2 C (a heterogeneous population with 2 C DNA content, consisting of spermatogonia and testicular somatic cells); LZ (leptotene and zygotene spermatocytes); PS (pachytene spermatocytes); and RS (round spermatids), totaling 12 libraries, i.e. four different cell populations, with three biological replicates each. As previously stated [42], the 2 C cell population was obtained from a testicular cell suspension of a pool of up to five individuals of 12–14 days *postpartum* (d*pp*), which excludes the possibility that this population contains spermatocytes II; LZ and PS cell populations were classified from 15 to 19 d*pp* animals, and RS from 22 to 24 d*pp* animals.

### General data processing

Neither the RNA extraction method nor the library type focused on small RNAs, and therefore the analysis was centered on mRNAs and lncRNAs. Moreover, only molecules ≥ 200 bp were considered in this study, and every genome unit that generated transcripts above that size, was considered a gene.

Low-quality reads (Q < 20) and adapter sequences were trimmed using TrimGalore [80]. Reads that passed quality control were mapped with HISAT2 (http://daehwankimlab.github.io/hisat2/), employing dta (downstream-transcriptome-assembly) parameters. We performed genome-guided alignment, using both paired and unpaired reads for each cell population, and discarding reads with multimapping. *Mus musculus* Ensembl database (Grcm38.92 release) was used as reference genome.

We used Strawberry [81] to assemble new transcripts under the guidance of genome alignment, employing 10 reads as minimum support per splice site, and per exon. Besides, during the set-up we used different depth cut-offs and found that the results did not substantially change. We therefore chose to work with a minimum of 10X coverage, as it turned out to be a strong support (Supplementary Figure S6). In order to generate a unique reference of our assemblies, we employed StringTie, with merge option [82].

A correlation matrix was constructed in R bioconductor (http://www.R-project.org), calculating Pearson’s correlation coefficient between FPKM expression of every transcript in each of the 12 samples, to appreciate the strength of the correlations between ourreplicates.

We analyzed transcripts discovery saturation throughout rarefaction curves at different read depths, with the aim of checking if we reached saturation in the 4 cell populations, and to rule out artifacts. For this purpose, we carried out counts with FeatureCounts [83] using the data from this paper, and compared them to those of da Cruz et al. [41]. (SRA repository access number PRJNA317251). The following conditions were used: -O assigns reads to all their overlapping meta-features; -S0 indicates unstranded reads; -t specifies feature type(s) in a GTF annotation; and -g states for attribute type in GTF annotation, with the reference that we previously generated. Subsequently, in R, we employed the function “estimate saturation” from the RNAseQC library [84], which allows cutting by depth and thus seeing how transcript detection occurs, based on the number of reads.

### Data comparison with single cell RNAseq studies

For comparison of our RNA lists with those from another report in which different spermatogenic stages were studied at scRNA-seq level [37], we downloaded the raw data from NCBI’s Gene Expression Omnibus (GEO) data repository (http://www.ncbi.nlm.nih.gov/gen/) with the accession ID: GSE107644. We mapped the raw data from that study with the same pipeline used for our own data, then performed the counts of our data and those of single seq with our assembly employing HTseq-counts [85], and the generated lists were normalized with limma package for R [86], using the function removeBatchEffect. A Principal Component Analysis (PCA) was generated by means of Seurat (that uses normalized log CPM [Counts Per Million] values as input) [87].

### Detection of splice variants, analysis of coding potential, annotation, and structural prediction of putative proteins

The generated reference GTF file containing our assembly was converted to a FASTA file by means of gffread [88]. We used this FASTA file as input for the different employed software packages, to categorize the new transcripts into coding or noncoding. For this categorization, we used four different software packages in parallel: TransDecoder [89], CPC2 [90], LncADeep [91], and CPAT [92], all of them with their default parameters. For further analysis, we proceeded with the intersection of the four software packages.

Venn diagrams were constructed using free Bioinformatics & Evolutionary Genomics software (http://bioinformatics.psb.ugent.be/webtools/Venn/).

We used rMATS software (http://rnaseq-mats.sourceforge.net/) with its default parameters, for the analysis of the different types of AS patterns. For the determination of the number of transcripts per gene for coding and noncoding transcripts, we plotted them normalized as the percentage of total transcripts in each category. T-test was conducted to calculate statistical values between the cell types using their replicates. We used PlotTranscripts function [82] to see the transcript structure and expression for single gene analysis.

With the aim of assessing the functionality of the unannotated genes, we conducted a primary annotation by means of Trinotate [93], using all the software´s available methods and databases (BLASTX using SWISSPROT, RNAMMER, prot_id, BLASTP, Pfam, SignalP, TMHMM, eggNOG, KEGG, Gene Ontology BLAST, Gene Ontology Pfam). Modeling of predicted proteins was conducted through Swiss-Model (https://swissmodel.expasy.org/interactive), and an analysis of putative protein domains was performed with Pfam (http://pfam-legacy.xfam.org).

### Differential gene expression analysis

Differential gene expression between the four testicular cell populations was obtained employing StringTie -e (quantification function) -B (output option for Ballgown analysis), --fr (stranded library fr-secondstrand), and using our assembly as a reference to generate the counts and FPKM.

Pairwise comparisons were made in chronological order of appearance along the first spermatogenic wave (LZ vs. 2 C; PS vs. LZ; RS vs. PS), by means of Ballgown software [82]. A log2 fold change (FC) ≥ 2 or ≤-2, and q value < 0.05 was used to filter the DE genes. We also filtered by a minimum of 10X coverage.

All followed bioinformatics protocols are illustrated in Fig. 2.

### Animals and Ethics statement

Animal procedures were performed following the recommendations of the Uruguayan National Commission of Animal Experimentation (CNEA, http://www.cnea.org.uy), approved experimental protocol 001/02/2012 (code: 008/11). Male CD-1 Swiss mice (*Mus musculus*) were obtained from the animal facility at Instituto de Higiene of Facultad de Medicina (UdelaR, Montevideo, Uruguay). Animals were euthanized by cervical dislocation, in accordance with the National Law of Animal Experimentation 18,611 (Uruguay). Immediately after euthanasia testes were dissected and tunica albuginea was removed, before proceeding to the preparation of testicular cell suspensions for sorting and RT-PCR.

### Confirmative RT-PCR

For the confirmation of the selected splice variants, we designed specific primers to amplify, either the newly identified transcripts or the annotated ones. Especially designed primers are listed in Supplementary Table S3.

Cell fractions containing 3,000 cells each from 2 C, LZ, PS, and RS populations were sorted as previously described [42]. Briefly, cell suspensions were prepared and stained with Vybrant DyeCycle Green (VDG; Invitrogen, Life Technologies, Carlsbad, CA), as instructed [45]. The sorting was conducted in a MoFlo Astrios EQ (Beckman Coulter) in Purify mode (with 1–2 drops). The sorted cell fractions were used for confirmative RT-PCR by means of the Power SYBR Green Cells-to-Ct Kit (Ambion-Life Technologies) essentially as instructed, using random primers for first strand cDNA synthesis. We used 2 µL cDNA in 20 µL final volume PCR reaction following the instructions of the Cells-to-Ct Kit, and employing a CFX96 Touch Real-Time PCR Detection System 1 (BioRad, Hercules, CA), with three biological replicas each. Although RT-qPCR was not mandatory for the confirmation of splice variants, we chose to use this kit for its high sensitivity, given the low input of sorted cells. The PCR reactions were run in conventional agarose gels and stained with GelRed (Biotium, Fremont, CA, USA).

### Electronic supplementary material

Below is the link to the electronic supplementary material.

**Supplementary Figure S1.** **Correlation matrix for the 4 cell populations with 3 biological replicas each.**

**Supplementary Figure S2.Principal component analysis (PCA) comparing our RNAseq data with those of a scRNA-seq of 20 different spermatogenic cell subtypes **[37]. The cell populations from our study are represented as squares, while those from the single-cell study are depicted as circles. Notably, the correlation is very good taking into consideration that many conditions in both experiments were different. As an example, in this single-cell study the spermatogenic process was manipulated through a combination of transgenic labeling and artificial synchronization of the cycle of the seminiferous epithelium, and therefore a slight shift in the time of appearance of some transcripts cannot be ruled out. Of mention, the data from our 2C cell population was not included for comparison, as besides spermatogonia it contains somatic testicular cells, which were not included in the single-cell study.L: leptotene; Z: zygotene; LZ: lepto/zygotene; eP: early pachytene; mP: medium pachytene; lP: late pachytene; PS: pachytene spermatocytes; D: diplotene; RS: round spermatids; RS2_1-5: early round spermatids, steps 1-2; RS8_1-5: late round spermatids, steps 7-8.

**Supplementary Figure S3.Genes and transcripts expressed in our lists.A)** Flow chart representing the process of categorizing the genes expressed in the four testicular cell populations, and the expressed transcripts generated from them. The categories are, in each case, annotated or unannotated, and, for the unannotated transcripts, high or low coding potential. The number of genes or transcripts in each category is indicated. It is important to recall that the number of categorized transcripts according to coding potential is only a subset, as we only kept the intersection of the four used software programs. The individual result of each program is shown at the bottom of the figure.**B-D)**Number of expressed genes and transcripts arising from them, discriminated by the four testicular cell populations. **B)** Pie chart of annotated genes (aG: blue) and unannotated genes (uG: red) expressed in each of the four cell populations that passed all the filters. **C)** Pie chart of annotated transcripts (aT: blue) and unannotated transcripts (uT: red) expressed in each of the four spermatogenic cell populations. **D)** Pie chart showing the origin of the unannotated transcripts in our lists for each of the four cell populations, either undisclosed splice variants of already annotated genes (of aG: blue), or transcripts arising from unannotated genes (of uG: red). Note that the unannotated genes and transcripts are more stage-specific than the annotated ones. As a consequence, the different cell populations share a higher number of annotated expressed genes/transcripts compared to the unannotated ones. Due to the transcripts in common, this is visualized as a higher proportion of annotated genes and transcripts when they are separately analyzed by cell population.

**Supplementary Figure S4. Transcript distribution in the four testicular cell populations. A) **Representation of the unannotated transcripts that were coincidentally identified as coding or noncoding with the four programs for coding potential analysis and depicted in Figure 5C, but distributed according to the different categories (*i.e. *coding or noncoding; splice variants of already annotated genes or transcripts of unannotated genes). **B) **Representation of all the 33,002 newly identified transcripts (previous to their filtration for coding potential), and showing 6,708 transcripts as expressed in 2C; 18,607 in LZ; 12,353 in PS; and 12,575 in RS. **C) **Representation of all the detected transcripts in our lists (both annotated and unannotated).

**Supplementary Figure S5. Saturation and expression distribution in the four cell populations.A)** Rarefaction analysis in the studied samples, including data of da Cruz *et al*., 2016 [41]. **B**) Histogram distribution analysis of expression in the four testicular cell populations. The values of the lowest expression range (corresponding to 2C: 85,263 transcripts; LZ: 68,740; PS: 84,947; and RS: 76,905), were excluded from the graph to have a clearer representation.

**Supplementary Figure S6.Semi-logarithmic plot of identified transcripts *vs* coverage for 7 different transcript abundance cut-offs.** The ordinate axis (RNA abundance) indicates the logarithmic scale (log2) of transcripts number.

**Supplementary Figure S7: Original agarose gels from Figure 7.** The cropped regions are demarcated by red squares.

**Supplementary Table S1: Expression and annotation of detected transcripts. **ENSMUST stands for Ensembl-annotated transcripts, while MSTRG designates unannotated transcripts.

**Supplementary Table S2: Expression and** **annotation of the 223 newly identified transcripts with high coding** **potential, that correspond to 191 unannotated genes.**

**Supplementary Table S** **3** **: List of the PCR primers used in this study.**

## Data Availability

The datasets used and analysed during the current study are available in the SRA repository, with access number PRJNA548952, (https://www.ncbi.nlm.nih.gov/sra/?term=PRJNA548952).

## Abbreviations

aG: Annotated genes
AS: Alternative splicing
aT: Annotated transcripts
A3SS: Alternative 3′ splice site
A5SS: Alternative 5′ splice site
d*pp*: Days *post**partum*
DE: Differentially expressed
lncRNAs: Long noncoding RNAs
LZ: Leptotene-zygotene
MXE: Mutually exclusive exons
PS: Pachytene spermatocytes
RI: Retained intron
RS: Round spermatids
scRNA-seq: Single-cell RNA sequencing
SE: Skipping exon
uG: Unannotated genes
uT: Unannotated transcripts

## References

[1] Kleene KC (2001). A possible meiotic function of the peculiar patterns of gene expression in mammalian spermatogenic cells. Mech Dev.

[2] Geisinger A, Rodríguez-Casuriaga R, Benavente R. Transcriptomics of meiosis in the male mouse. Front Cell Dev Biol. 2021;9. 10.3389/fcell.2021.626020 PMC7973102 33748111

[3] Green CD, Ma Q, Manske GL, Shami AN, Zheng X, Marini S (2018). A Comprehensive Roadmap of Murine Spermatogenesis defined by single-cell RNA-Seq. Dev Cell.

[4] Melé M, Ferreira PG, Reverter F, DeLuca DS, Monlong J, Sammeth M (2015). Human genomics. The human transcriptome across tissues and individuals. Science.

[5] Uhlén M, Fagerberg L, Hallström BM, Lindskog C, Oksvold P, Mardinoglu A (2015). Proteomics. Tissue-based map of the human proteome. Science.

[6] Soumillon M, Necsulea A, Weier M, Brawand D, Zhang X, Gu H (2013). Cellular source and mechanisms of high transcriptome complexity in the mammalian testis. Cell Rep.

[7] Cabili M, Trapnell C, Goff L, Koziol M, Tazon-Vega B, Regev A (2011). Integrative annotation of human large intergenic noncoding RNAs reveals global properties and specific subclasses. Genes Dev.

[8] Darbellay F, Necsulea A (2020). Comparative transcriptomics analyses across species, organs, and Developmental stages Reveal functionally constrained lncRNAs. Mol Biol Evol.

[9] Derrien T, Johnson R, Bussotti G, Tanzer A, Djebali S, Tilgner H (2012). The GENCODE v7 catalog of human long noncoding RNAs: analysis of their gene structure, evolution, and expression. Genome Res.

[10] Hong SH, Kwon JT, Kim J, Jeong J, Kim J, Lee S et al. Profiling of testis-specific long noncoding RNAs in mice. BMC Genomics. 2018;19(1). 10.1186/s12864-018-4931-3 PMC6048885 30012089

[11] Necsulea A, Soumillon M, Warnefors M, Liechti A, Daish T, Zeller U (2014). The evolution of lncRNA repertoires and expression patterns in tetrapods. Nature.

[12] Bortvin A (2013). PIWI-interacting RNAs (piRNAs) - a mouse testis perspective. Biochem (Mosc).

[13] de Mateo S, Sassone-Corsi P (2014). Regulation of spermatogenesis by small non-coding RNAs: role of the germ granule. Semin Cell Dev Biol.

[14] Kotaja N (2014). MicroRNAs and spermatogenesis. Fertil Steril.

[15] Yadav RP, Kotaja N (2014). Small RNAs in spermatogenesis. Mol Cell Endocrinol.

[16] Hilz S, Modzelewski AJ, Cohen PE, Grimson A (2016). The roles of microRNAs and siRNAs in mammalian spermatogenesis. Development.

[17] He C, Wang K, Gao Y, Wang C, Li L, Liao Y et al. Roles of noncoding RNA in Reproduction. Front Genet. 2021;12. 10.3389/fgene.2021.777510 PMC8695933 34956326

[18] Yeo G, Holste D, Kreiman G, Burge CB. Variation in alternative splicing across human tissues. Genome Biol. 2004;5(10). 10.1186/gb-2004-5-10-r74 PMC545594 15461793

[19] Kan Z, Garrett-Engele PW, Johnson JM, Castle JC (2005). Evolutionarily conserved and diverged alternative splicing events show different expression and functional profiles. Nucleic Acids Res.

[20] Naro C, Cesari E, Sette C (2021). Splicing regulation in brain and testis: common themes for highly specialized organs. Cell Cycle.

[21] Mazin PV, Khaitovich P, Cardoso-Moreira M, Kaessmann H (2021). Alternative splicing during mammalian organ development. Nat Genet.

[22] Legrand JMD, Hobbs RM (2018). RNA processing in the male germline: mechanisms and implications for fertility. Semin Cell Dev Biol.

[23] Song H, Wang L, Chen D, Li F (2020). The function of Pre-mRNA Alternative Splicing in Mammal Spermatogenesis. Int J Biol Sci.

[24] Idler RK, Yan W (2012). Control of messenger RNA fate by RNA-binding proteins: an emphasis on mammalian spermatogenesis. J Androl.

[25] Licatalosi DD (2016). Roles of RNA-binding proteins and post-transcriptional regulation in driving male germ cell development in the mouse. Adv Exp Med Biol.

[26] MacDonald CC. Tissue-specific mechanisms of alternative polyadenylation: Testis, brain, and beyond (2018 update). Wiley Interdiscip Rev RNA. 2019;10(4). 10.1002/wrna.1526 PMC6617714 30816016

[27] Grosso AR, Gomes AQ, Barbosa-Morais NL, Caldeira S, Thorne NP, Grech G (2008). Tissue-specific splicing factor gene expression signatures. Nucleic Acids Res.

[28] de la Grange P, Gratadou L, Delord M, Dutertre M, Auboeuf D (2010). Splicing factor and exon profiling across human tissues. Nucleic Acids Res.

[29] Wu D, Khan FA, Huo L, Sun F, Huang C (2022). Alternative splicing and MicroRNA: epigenetic mystique in male reproduction. RNA Biol.

[30] Bao J, Tang C, Li J, Zhang Y, Bhetwal BP, Zheng H et al. RAN-binding protein 9 is involved in alternative splicing and is critical for male germ cell development and male fertility. PLoS Genet. 2014;10(12). 10.1371/journal.pgen.1004825 PMC4256260 25474150

[31] Iwamori N, Tominaga K, Sato T, Riehle K, Iwamori T, Ohkawa Y (2016). MRG15 is required for pre-mRNA splicing and spermatogenesis. Proc Natl Acad Sci U S A.

[32] Hannigan MM, Zagore LL, Licatalosi DD (2017). Ptbp2 controls an alternative splicing network required for cell communication during spermatogenesis. Cell Rep.

[33] Laiho A, Kotaja N, Gyenesei A, Sironen A. Transcriptome profiling of the murine testis during the first wave of spermatogenesis. PLoS ONE. 2013;8(4). 10.1371/journal.pone.0061558 PMC3629203 23613874

[34] Schmid R, Grellscheid SN, Ehrmann I, Dalgliesh C, Danilenko M, Paronetto MP (2013). The splicing landscape is globally reprogrammed during male meiosis. Nucleic Acids Res.

[35] Margolin G, Khil PP, Kim J, Bellani MA, Camerini-Otero RD. Integrated transcriptome analysis of mouse spermatogenesis. BMC Genomics. 2014;15(1). 10.1186/1471-2164-15-39 PMC3906902 24438502

[36] Zuo H, Zhang J, Zhang L, Ren X, Chen X, Hao H et al. Transcriptomic variation during spermiogenesis in mouse germ cells. PLoS ONE. 2016;11(11). 10.1371/journal.pone.0164874 PMC5105947 27835637

[37] Chen Y, Zheng Y, Gao Y, Lin Z, Yang S, Wang T (2018). Single-cell RNA-seq uncovers dynamic processes and critical regulators in mouse spermatogenesis. Cell Res.

[38] Li Q, Li T, Xiao X, Ahmad DW, Zhang N, Li H (2020). Specific expression and alternative splicing of mouse genes during spermatogenesis. Mol Omics.

[39] Chalmel F, Lardenois A, Evrard B, Rolland AD, Sallou O, Dumargne MC et al. High-resolution profiling of novel transcribed regions during rat spermatogenesis. Biol Reprod. 2014;91(1). 10.1095/biolreprod.114.118166 24740603

[40] Rolland AD, Evrard B, Darde TA, Le Beguec C, Le Bras Y, Bensalah K (2019). RNA profiling of human testicular cells identifies syntenic lncRNAs associated with spermatogenesis. Hum Reprod.

[41] da Cruz I, Rodríguez-Casuriaga R, Santiñaque FF, Farías J, Curti G, Capoano CA et al. Transcriptome analysis of highly purified mouse spermatogenic cell populations: gene expression signatures switch from meiotic-to postmeiotic-related processes at pachytene stage. BMC Genomics. 2016;17(1). 10.1186/s12864-016-2618-1 PMC4837615 27094866

[42] Trovero MF, Rodríguez-Casuriaga R, Romeo C, Santiñaque FF, François M, Folle GA (2020). Revealing stage-specific expression patterns of long noncoding RNAs along mouse spermatogenesis. RNA Biol.

[43] Rodríguez-Casuriaga R, Folle GA, Santiñaque F, López-Carro B, Geisinger A. Simple and efficient technique for the preparation of testicular cell suspensions. J Visualized Experiments. 2013;(78):1–7. 10.3791/50102 PMC3846780 23963251

[44] Rodríguez-Casuriaga R, Santiñaque FF, Folle GA, Souza E, López-Carro B, Geisinger A (2014). Rapid preparation of rodent testicular cell suspensions and spermatogenic stages purification by flow cytometry using a novel blue-laser-excitable vital dye. MethodsX.

[45] Geisinger A, Rodríguez-Casuriaga R (2017). Flow cytometry for the isolation and characterization of rodent meiocytes. Methods Mol Biol.

[46] Liu W, Wang F, Xu Q, Shi J, Zhang X, Lu X et al. BCAS2 is involved in alternative mRNA splicing in spermatogonia and the transition to meiosis. Nat Commun. 2017;8. 10.1038/ncomms14182 PMC5290162 28128212

[47] Rabbani M, Zheng X, Manske GL, Vargo A, Shami AN, Li JZ (2022). Decoding the spermatogenesis program: new insights from transcriptomic analyses. Annu Rev Genet.

[48] Monesi V (1964). Ribonucleic acid synthesis during mitosis and meiosis in the mouse testis. J Cell Biol.

[49] Kierszenbaum AL, Tres LL (1974). Nucleolar and perichromosomal RNA synthesis during meiotic prophase in the mouse testis. J Cell Biol.

[50] Page J, De La Fuente R, Manterola M, Parra MT, Viera A, Berríos S (2012). Inactivation or non-reactivation: what accounts better for the silence of sex chromosomes during mammalian male meiosis?. Chromosoma.

[51] Tilgner H, Knowles DG, Johnson R, Davis CA, Chakrabortty S, Djebali S (2012). Deep sequencing of subcellular RNA fractions shows splicing to be predominantly co-transcriptional in the human genome but inefficient for lncRNAs. Genome Res.

[52] Gill ME, Rohmer A, Erkek-Ozhan S, Liang CY, Chun S, Ozonov EA, Peters AHFM (2023). De novo transcriptome assembly of mouse male germ cells reveals novel genes, stage-specific bidirectional promoter activity, and noncoding RNA expression. Genome Res.

[53] Naro C, Jolly A, Di Persio S, Bielli P, Setterblad N, Alberdi AJ (2017). An orchestrated intron retention program in meiosis controls timely usage of transcripts during germ cell differentiation. Dev Cell.

[54] Anguera MC, Ma W, Clift D, Namekawa S, Kelleher RJ, Lee JT. Tsx produces a long noncoding RNA and has general functions in the germline, stem cells, and brain. PLoS Genet. 2011;7(9). 10.1371/journal.pgen.1002248 PMC3164691 21912526

[55] Ni MJ, Hu ZH, Liu Q, Liu MF, Lu MH, Zhang JS et al. Identification and characterization of a novel non-coding RNA involved in sperm maturation. PLoS ONE. 2011;6(10). 10.1371/journal.pone.0026053 PMC3192136 22022505

[56] Lü M, Tian H, Cao YX, He X, Chen L, Song X et al. Downregulation of miR-320a/383-sponge-like long non-coding RNA NLC1-C (narcolepsy candidate-region 1 genes) is associated with male infertility and promotes testicular embryonal carcinoma cell proliferation. Cell Death Dis. 2015;6(11). 10.1038/cddis.2015.267 PMC4670917 26539909

[57] Li L, Wang M, Wang M, Wu X, Geng L, Xue Y et al. A long non-coding RNA interacts with Gfra1 and maintains survival of mouse spermatogonial stem cells. Cell Death Dis 2016;7(3). 10.1038/cddis.2016.24 PMC4823932 26962690

[58] Kataruka S, Akhade VS, Kayyar B, Rao MRS. Mrhl Long noncoding RNA mediates meiotic commitment of mouse spermatogonial cells by regulating Sox8 expression. Mol Cell Biol. 2017;37(14). 10.1128/MCB.00632-16 PMC5492173 28461394

[59] Nakajima R, Sato T, Ogawa T, Okano H, Noce T. A noncoding RNA containing a SINE-B1 motif associates with meiotic metaphase chromatin and has an indispensable function during spermatogenesis. PLoS ONE. 2017;12(6). 10.1371/journal.pone.0179585 PMC5489172 28658256

[60] Li W, Ning JZ, Cheng F, Yu WM, Rao T, Ruan Y (2018). MALAT1 promotes cell apoptosis and suppresses cell proliferation in testicular ischemia-reperfusion injury by sponging MiR-214 to modulate TRPV4 expression. Cell Physiol Biochem.

[61] Joshi M, Rajender S. Long non-coding RNAs (lncRNAs) in spermatogenesis and male infertility. Reprod Biol Endocrinol. 2020;18(1). 10.1186/s12958-020-00660-6 PMC7599102 33126901

[62] Li K, Xu J, Luo Y, Zou D, Han R, Zhong S (2021). Panoramic transcriptome analysis and functional screening of long noncoding RNAs in mouse spermatogenesis. Genome Res.

[63] Liu W, Zhao Y, Liu X, Zhang X, Ding J, Li Y et al. A novel meiosis-related lncRNA, Rbakdn, contributes to spermatogenesis by stabilizing Ptbp2. Front Genet. 2021;12. 10.3389/fgene.2021.752495 PMC8542969 34707642

[64] Harfe BD, Jinks-Robertson S (2000). DNA mismatch repair and genetic instability. Annu Rev Genet.

[65] Edelmann W, Cohen PE, Kneitz B, Winand N, Lia M, Heyer J (1999). Mammalian MutS homologue 5 is required for chromosome pairing in meiosis. Nat Genet.

[66] Milano CR, Kim Holloway J, Zhang Y, Jin B, Smith C, Bergman A (2019). Mutation of the ATPase domain of MutS Homolog-5 (MSH5) reveals a requirement for a functional MutSγ complex for all crossovers in mammalian meiosis. G3. (Bethesda).

[67] Wu HY, Wei P, Morgan JI. Role of Cytosolic Carboxypeptidase 5 in neuronal survival and spermatogenesis. Sci Rep. 2017;7. 10.1038/srep41428 PMC5269731 28128286

[68] Giordano T, Gadadhar S, Bodakuntla S, Straub J, Leboucher S, Martinez G et al. Loss of the deglutamylase CCP5 perturbs multiple steps of spermatogenesis and leads to male infertility. J Cell Sci. 2019;132(3). 10.1242/jcs.226951 30635446

[69] Fonseca BD, Lahr RM, Damgaard CK, Alain T, Berman AJ. LARP1 on TOP of ribosome production. Wiley Interdiscip Rev RNA. 2018;9(5). 10.1002/wrna.1480 PMC6214789 29722158

[70] Berman AJ, Thoreen CC, Dedeic Z, Chettle J, Roux PP, Blagden SP (2021). Controversies around the function of LARP1. RNA Biol.

[71] Bao J, Wang L, Lei J, Hu Y, Liu Y, Shen H (2012). STK31(TDRD8) is dynamically regulated throughout mouse spermatogenesis and interacts with MIWI protein. Histochem Cell Biol.

[72] Zhong L, Liu J, Hu Y, Wang W, Xu F, Xu W (2017). STK31 as novel biomarker of metastatic potential and tumorigenicity of colorectal cancer. Oncotarget.

[73] Xiong J, Xing S, Dong Z, Niu L, Xu Q, Liu P (2020). STK31 regulates the proliferation and cell cycle of lung cancer cells via the Wnt/βcatenin pathway and feedback regulation by cmyc. Oncol Rep.

[74] Bae DH, Kim HJ, Yoon BH, Park JL, Kim M, Kim SK et al. STK31 upregulation is associated with chromatin remodeling in gastric cancer and induction of tumorigenicity in a xenograft mouse model. Oncol Rep. 2021;45(4). 10.3892/or.2021.7993 PMC7934220 33649810

[75] Dowdle JA, Mehta M, Kass EM, Vuong BQ, Inagaki A, Egli D et al. Mouse BAZ1A (ACF1) is dispensable for double-strand break repair but is essential for averting improper gene expression during spermatogenesis. PLoS Genet. 2013;9(11). 10.1371/journal.pgen.1003945 PMC3820798 24244200

[76] Yadav RP, Leskinen S, Ma L, Mäkelä JA, Kotaja N (2022). Chromatin remodelers HELLS, WDHD1 and BAZ1A are dynamically expressed during mouse spermatogenesis. Reproduction.

[77] Tsuchida J, Nishina Y, Wakabayashi N, Nozaki M, Sakai Y, Nishimune Y (1998). Molecular cloning and characterization of meichroacidin (male meiotic metaphase chromosome-associated acidic protein). Dev Biol.

[78] Zheng W, Li F, Ding Z, Liu H, Zhu L, Xu C et al. Distinct architecture and composition of mouse axonemal radial spoke head revealed by cryo-EM. Proc Natl Acad Sci U S A. 2021;118(4). 10.1073/pnas.2021180118 PMC7848523 34871179

[79] Kott E, Legendre M, Copin B, Papon JF, Dastot-Le Moal F, Montantin G (2013). Loss-of-function mutations in RSPH1 cause primary ciliary dyskinesia with central-complex and radial-spoke defects. Am J Hum Genet.

[80] Lindgreen S. AdapterRemoval: easy cleaning of next-generation sequencing reads. BMC Res Notes. 2012;5. 10.1186/1756-0500-5-337 PMC3532080 22748135

[81] Liu R, Dickerson J, Strawberry. Fast and accurate genome-guided transcript reconstruction and quantification from RNA-Seq. PLoS Comput Biol. 2017;13(11). 10.1371/journal.pcbi.1005851 PMC5720828 29176847

[82] Pertea M, Kim D, Pertea GM, Leek JT, Salzberg SL (2016). Transcript-level expression analysis of RNA-seq experiments with HISAT, StringTie and Ballgown. Nat Protoc.

[83] Liao Y, Smyth GK, Shi W (2014). featureCounts: an efficient general purpose program for assigning sequence reads to genomic features. Bioinformatics.

[84] Deluca DS, Levin JZ, Sivachenko A, Fennell T, Nazaire MD, Williams C (2012). RNA-SeQC: RNA-seq metrics for quality control and process optimization. Bioinformatics.

[85] Anders S, Pyl PT, Huber W (2015). HTSeq–a Python framework to work with high-throughput sequencing data. Bioinformatics.

[86] Ritchie ME, Phipson B, Wu D, Hu Y, Law CW, Shi W (2015). Limma powers differential expression analyses for RNA-sequencing and microarray studies. Nucleic Acids Res.

[87] Hao Y, Hao S, Andersen-Nissen E, Mauck WM, Zheng S, Butler A (2021). Integrated analysis of multimodal single-cell data. Cell.

[88] Pertea G, Pertea M. GFF utilities: GffRead and GffCompare. F1000Res. 2020;9. 10.12688/f1000research.23297.1 PMC7222033 32489650

[89] Tang S, Lomsadze A, Borodovsky M. Identification of protein coding regions in RNA transcripts. Nucleic Acids Res. 2015;43(12). 10.1093/nar/gkv227 PMC4499116 25870408

[90] Kang YJ, Yang DC, Kong L, Hou M, Meng YQ, Wei L (2017). CPC2: a fast and accurate coding potential calculator based on sequence intrinsic features. Nucleic Acids Res.

[91] Yang C, Yang L, Zhou M, Xie H, Zhang C, Wang MD (2018). LncADeep: an ab initio lncRNA identification and functional annotation tool based on deep learning. Bioinformatics.

[92] Wang L, Park HJ, Dasari S, Wang S, Kocher JP, Li W. CPAT: Coding-Potential Assessment Tool using an alignment-free logistic regression model. Nucleic Acids Res. 2013;41(6). 10.1093/nar/gkt006 PMC3616698 23335781

[93] Bryant DM, Johnson K, DiTommaso T, Tickle T, Couger MB, Payzin-Dogru D (2017). A tissue-mapped Axolotl De Novo Transcriptome enables identification of limb regeneration factors. Cell Rep.

[94] Rodríguez-Casuriaga R, Geisinger A. Contributions of Flow Cytometry to the Molecular Study of Spermatogenesis in mammals. Int J Mol Sci. 2021;22(3):1151. 10.3390/ijms22031151 10.3390/ijms22031151 PMC7865295 33503798

