# Supplementary figures and images for: Uncovering a multitude of stage-specific splice variants and putative protein isoforms generated along mouse spermatogenesis
Source: BMC Genomics. 2024 Mar 20;25:295. doi: 10.1186/s12864-024-10170-z (PMC10953240; doi:10.1186/s12864-024-10170-z)

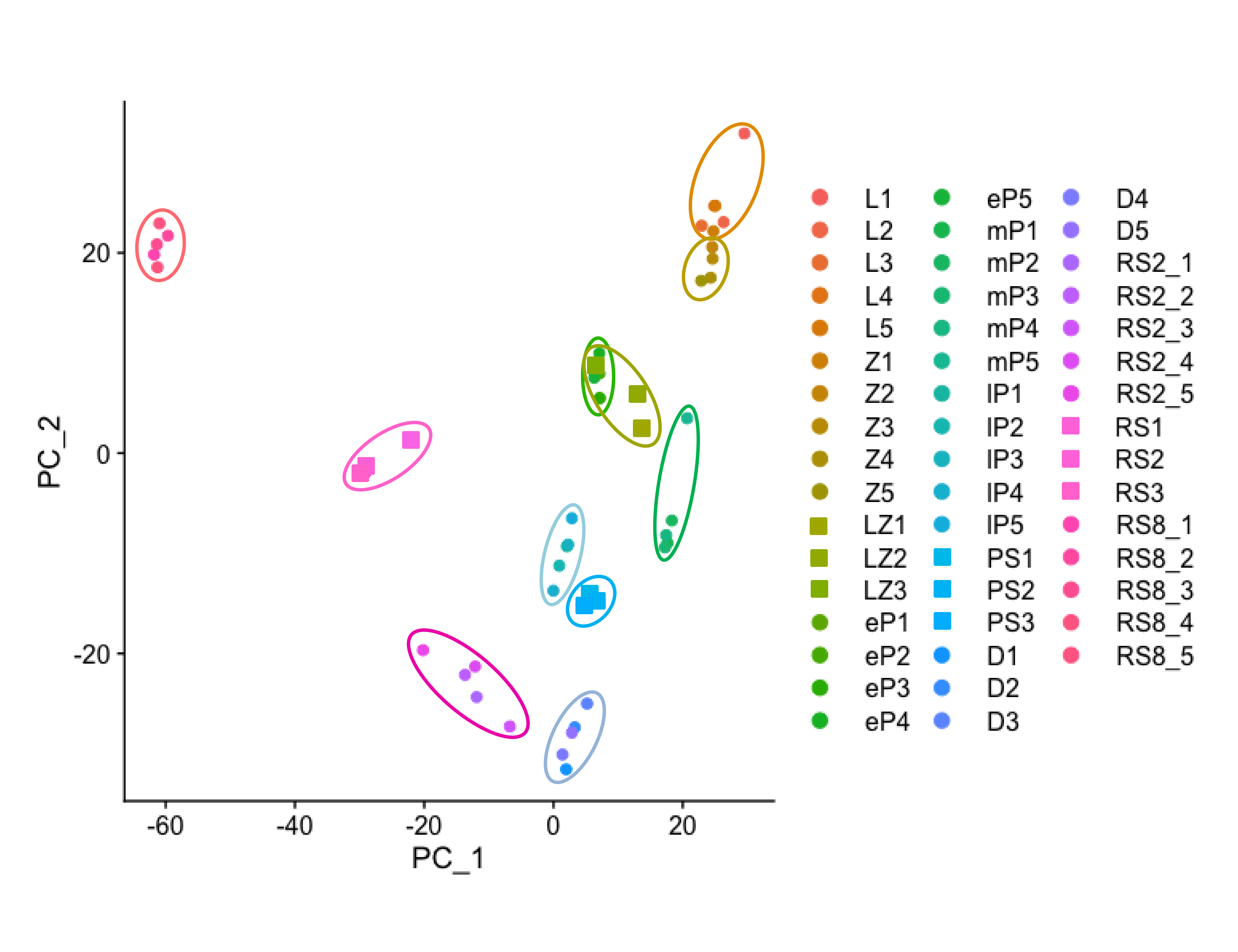

Supplement: Supplementary file 2 — Supplementary Figure S2.Principal component analysis (PCA) comparing our RNAseq data with those of a scRNA-seq of 20 different spermatogenic cell subtypes [37]. The cell populations from our study are represented as squares, while those from the single-cell study are depicted as circles. Notably, the correlation is very good taking into consideration that many conditions in both experiments were different. As an example, in this single-cell study the spermatogenic process was manipulated through a combination of transgenic labeling and artificial synchronization of the cycle of the seminiferous epithelium, and therefore a slight shift in the time of appearance of some transcripts cannot be ruled out. Of mention, the data from our 2C cell population was not included for comparison, as besides spermatogonia it contains somatic testicular cells, which were not included in the single-cell study.L: leptotene; Z: zygotene; LZ: lepto/zygotene; eP: early pachytene; mP: medium pachytene; lP: late pachytene; PS: pachytene spermatocytes; D: diplotene; RS: round spermatids; RS2_1-5: early round spermatids, steps 1-2; RS8_1-5: late round spermatids, steps 7-8. [file 12864_2024_10170_MOESM2_ESM.png]

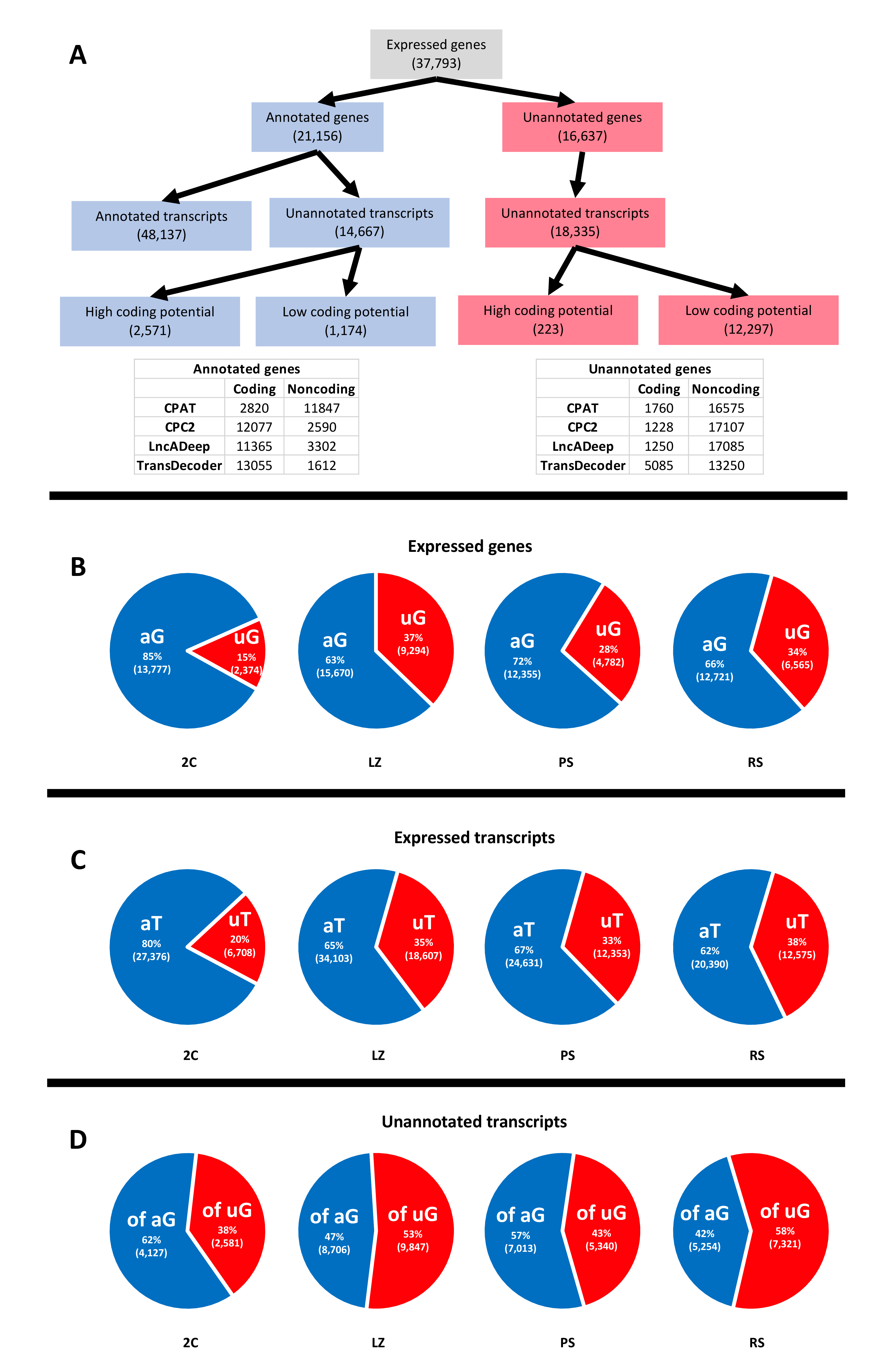

Supplement: Supplementary file 3 — Supplementary Figure S3.Genes and transcripts expressed in our lists.A) Flow chart representing the process of categorizing the genes expressed in the four testicular cell populations, and the expressed transcripts generated from them. The categories are, in each case, annotated or unannotated, and, for the unannotated transcripts, high or low coding potential. The number of genes or transcripts in each category is indicated. It is important to recall that the number of categorized transcripts according to coding potential is only a subset, as we only kept the intersection of the four used software programs. The individual result of each program is shown at the bottom of the figure.B-D)Number of expressed genes and transcripts arising from them, discriminated by the four testicular cell populations. B) Pie chart of annotated genes (aG: blue) and unannotated genes (uG: red) expressed in each of the four cell populations that passed all the filters. C) Pie chart of annotated transcripts (aT: blue) and unannotated transcripts (uT: red) expressed in each of the four spermatogenic cell populations. D) Pie chart showing the origin of the unannotated transcripts in our lists for each of the four cell populations, either undisclosed splice variants of already annotated genes (of aG: blue), or transcripts arising from unannotated genes (of uG: red). Note that the unannotated genes and transcripts are more stage-specific than the annotated ones. As a consequence, the different cell populations share a higher number of annotated expressed genes/transcripts compared to the unannotated ones. Due to the transcripts in common, this is visualized as a higher proportion of annotated genes and transcripts when they are separately analyzed by cell population. [file 12864_2024_10170_MOESM3_ESM.png]

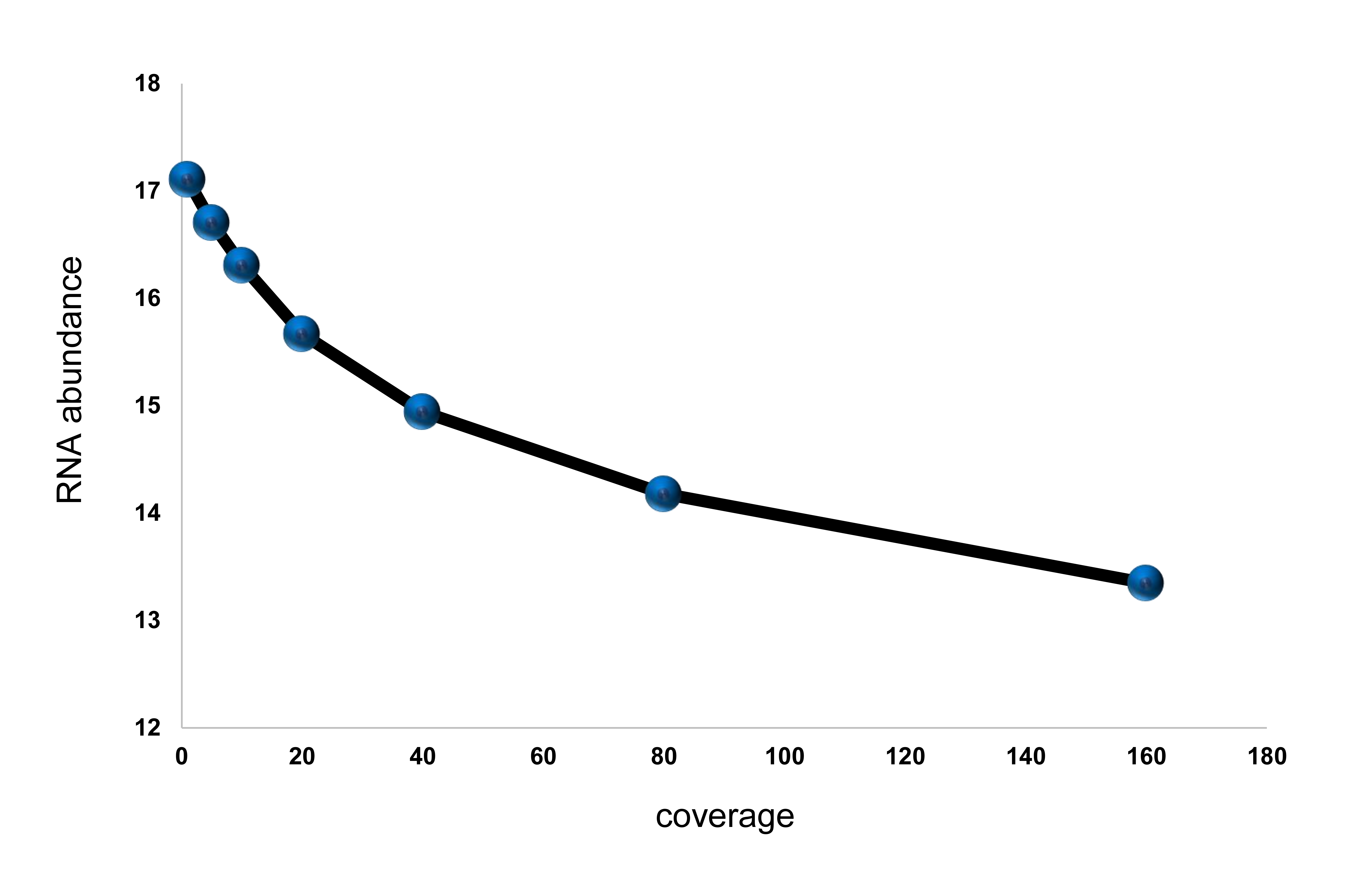

Supplement: Supplementary file 6 — Supplementary Figure S6.Semi-logarithmic plot of identified transcripts vs coverage for 7 different transcript abundance cut-offs. The ordinate axis (RNA abundance) indicates the logarithmic scale (log2) of transcripts number. [file 12864_2024_10170_MOESM6_ESM.png]

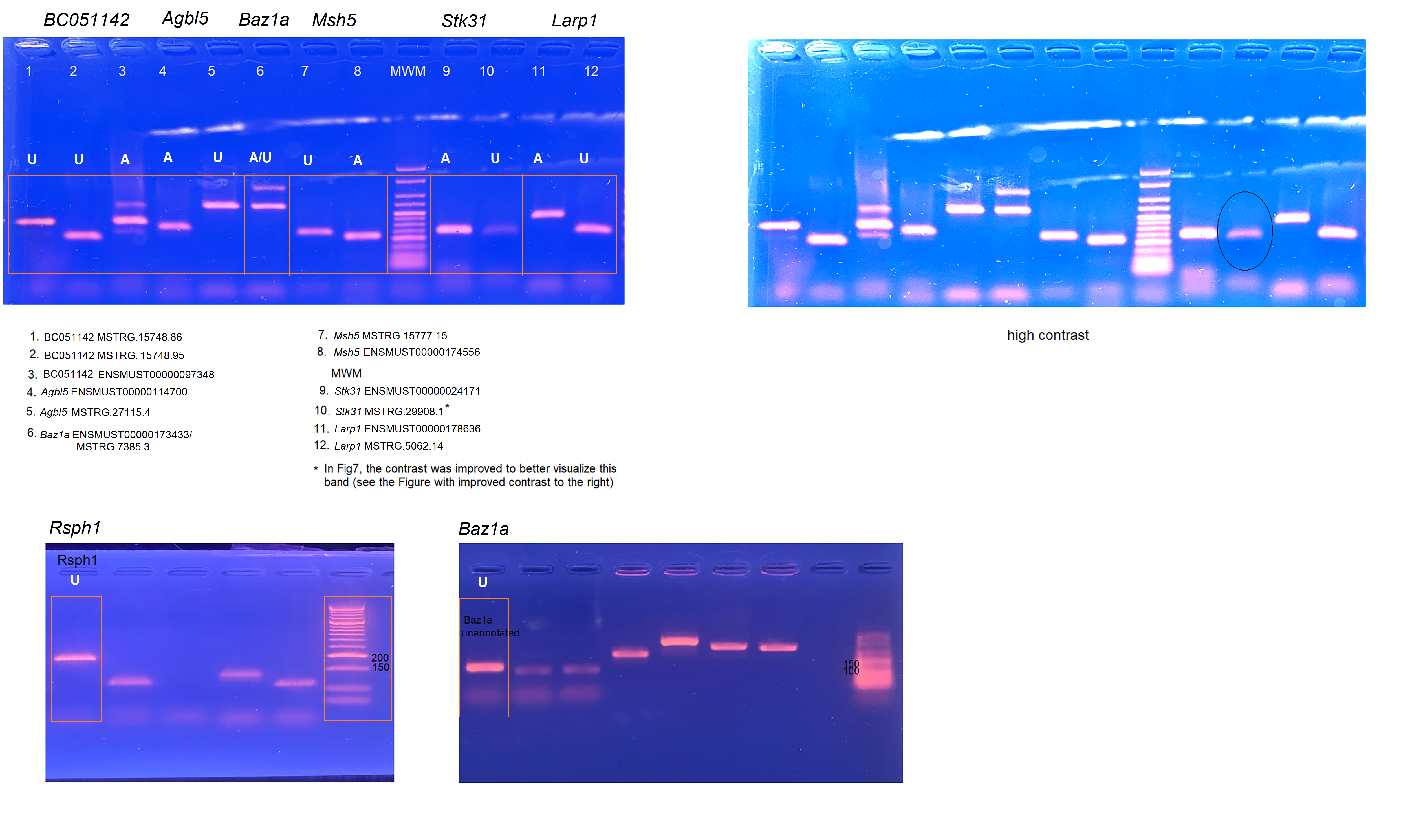

Supplement: Supplementary file 7 — Supplementary Figure S7: Original agarose gels from Figure 7. The cropped regions are demarcated by red squares. [file 12864_2024_10170_MOESM7_ESM.png]
